# Anti-Ku + myositis: an acquired inflammatory protein-aggregate myopathy

**DOI:** 10.1007/s00401-024-02765-3

**Published:** 2024-07-16

**Authors:** Marie-Therese Holzer, Akinori Uruha, Andreas Roos, Andreas Hentschel, Anne Schänzer, Joachim Weis, Kristl G. Claeys, Benedikt Schoser, Federica Montagnese, Hans-Hilmar Goebel, Melanie Huber, Sarah Léonard-Louis, Ina Kötter, Nathalie Streichenberger, Laure Gallay, Olivier Benveniste, Udo Schneider, Corinna Preusse, Martin Krusche, Werner Stenzel

**Affiliations:** 1 Division of Rheumatology and Systemic Inflammatory Diseases, III, Department of Medicine, University Medical Center Hamburg-Eppendorf, Martinistraße 52, 20246 Hamburg, Germany; 2 Department of Neuropathology, Charité. Universitätsmedizin Berlin, Corporate Member of Freie Universität Berlin and Humboldt-Universität zu Berlin, Berlin, Germany; 3 Department of Rheumatology, Charité, Universitätsmedizin Berlin, Corporate Member of Freie Universität Berlin and Humboldt-Universität zu Berlin, Berlin, Germany; 4 Department of Neurology, Tokyo Metropolitan Neurological Hospital, Tokyo, Japan; 5 Department of Neuropediatrics, Developmental Neurology and Social Pediatrics, Centre for Neuromuscular Disorders in Children, University Children’s Hospital Essen, University of Duisburg-Essen, Essen, Germany; 6 Department of Neurology, Medical Faculty, Heinrich Heine University Dusseldorf, 40225 Dusseldorf, Germany; 7 Brain and Mind Research Institute, Children’s Hospital of Eastern Ontario Research Institute, Ottawa, ON K1H 8L1 Canada; 8 Leibniz-Institut für Analytische Wissenschaften -ISAS- E.V., Dortmund, Germany; 9 Institute of Neuropathology, Justus-Liebig-University, Gießen, Germany; 10 Medical Faculty, Institute of Neuropathology, RWTH Aachen University, Aachen, Germany; 11 Department of Neurology, University Hospitals Leuven, Leuven, Belgium; 12 Department of Neurosciences, Laboratory for Muscle Diseases and Neuropathies, KU Leuven, and Leuven Brain Institute (LBI), Leuven, Belgium; 13 Department of Neurology, Friedrich-Baur-Institute, Ludwig-Maximilians-University, Munich, Germany; 14 Department for Rheumatology, Campus Kerckhoff of Justus-Liebig University Gießen, Bad Nauheim, Germany; 15 Reference Center of Neuromuscular Pathology Paris-Est, Pitié-Salpêtrière University Hospital, Paris, France; 16 Neuropathologie, Groupement Hospitalier Est, Hospices Civils de Lyon, Université Claude Bernard Lyon 1, Institut NeuroMyogène CNRS UMR 5261- INSERM U1315, Lyon, France; 17 Department of Internal Medicine, Edouard Herriot University Hospital, Hospices Civils de Lyon, Lyon, France; 18 Department of Internal Medicine and Clinical Immunology, Assistance Publique Hôpitaux de Paris, Sorbonne Université, Pitié-Salpêtrière University Hospital, Paris, France

**Keywords:** Autoantibodies, Myositis, Connective tissue diseases, Systemic sclerosis, Autophagy, Overlap syndrome

## Abstract

Myositis with anti-Ku-autoantibodies is a rare inflammatory myopathy associated with various connective tissue diseases. Histopathological studies have identified inflammatory and necrotizing aspects, but a precise morphological analysis and pathomechanistic disease model are lacking. We therefore aimed to carry out an in-depth morpho-molecular analysis to uncover possible pathomechanisms. Muscle biopsy specimens from 26 patients with anti-Ku-antibodies and unequivocal myositis were analyzed by immunohistochemistry, immunofluorescence, transcriptomics, and proteomics and compared to biopsy specimens of non-disease controls, immune-mediated necrotizing myopathy (IMNM), and inclusion body myositis (IBM). Clinical findings and laboratory parameters were evaluated retrospectively and correlated with morphological and molecular features. Patients were mainly female (92%) with a median age of 56.5 years. Isolated myositis and overlap with systemic sclerosis were reported in 31%, respectively. Isolated myositis presented with higher creatine kinase levels and cardiac involvement (83%), whereas systemic sclerosis-overlap patients often had interstitial lung disease (57%). Histopathology showed a wide spectrum from mild to pronounced myositis with diffuse sarcolemmal MHC-class I (100%) and -II (69%) immunoreactivity, myofiber necrosis (88%), endomysial inflammation (85%), thickened capillaries (84%), and vacuoles (60%). Conspicuous sarcoplasmic protein aggregates were p62, BAG3, myotilin, or immunoproteasomal beta5i-positive. Proteomic and transcriptomic analysis identified prominent up-regulation of autophagy, proteasome, and hnRNP-related cell stress. To conclude, Ku + myositis is morphologically characterized by myofiber necrosis, MHC-class I and II positivity, variable endomysial inflammation, and distinct protein aggregation varying from IBM and IMNM, and it can be placed in the spectrum of scleromyositis and overlap myositis. It features characteristic sarcoplasmic protein aggregation on an acquired basis being functionally associated with altered chaperone, proteasome, and autophagy function indicating that Ku + myositis exhibit aspects of an acquired inflammatory protein-aggregate myopathy.

**Supplementary Information:**

The online version contains supplementary material available at 10.1007/s00401-024-02765-3.

## Introduction

Anti-Ku-autoantibodies are myositis-associated antibodies (MAA) [5, 10, 37]. Ku protein is a heterodimer consisting of a 70K and 80K subunit (Ku70 and Ku80, respectively). It is associated with nuclear DNA by repairing double-strand breaks as well as by preventing telomere degradation [22, 53, 54].

The anti-Ku-antibody was first described in 1981 by Mimori et al. and was suggested to be a valuable marker for polymyositis-scleroderma-overlap-syndromes [42]. By now, it is known that these antibodies are associated with a wide spectrum of connective tissue diseases and rheumatoid arthritis. Nevertheless, anti-Ku + myositis remains a rare condition. Depending on the population, the prevalence of anti-Ku-autoantibodies in inflammatory myopathies ranges from 0.7 to 23% [7, 10, 16].

Anti-Ku-associated myositis can occur in various connective tissue diseases, especially in systemic lupus erythematosus and systemic sclerosis [48, 73] and overlap syndromes [17]. Apart from isolated myositis [17], patients may also present with different organ manifestations like cardiac involvement or skin involvement, arthritis, and interstitial lung disease (ILD), which seem to differ among the associated connective tissue diseases [16, 17, 55, 66]. A study showed that initial muscle weakness might not be necessarily present, but can develop throughout the disease. Interestingly, patients can also exhibit distal muscle weakness [16].

Histopathologically, smaller case studies identified Ku + myositis as inflammatory myopathy with necrotic aspects [55] or as mainly necrotic with only little inflammation [39]. Although results vary, Ku + myositis was compared to be “immune-mediated-necrotizing myopathy (IMNM)-like” due to the presence of necrotic myofibers [68].

Recently, immunostaining-based studies of autophagy markers like p62 in IMNM and inclusion body myositis (IBM) unveiled different staining patterns linking different types of autophagy to these diseases [14, 24]. One possible mechanism of action in autophagy is chaperone-assisted selective autophagy (CASA), in which a chaperone complex interacts with autophagy proteins to degrade proteins [4]. Especially in IMNM, the chaperone-guided autophagy involving BAG3 and Hsp70, among others, plays a central role in pathogenesis [24]. Furthermore, *BAG3* mutations can cause myofibrillar myopathies and cardiomyopathy, in which accumulation of BAG3 or myofibrillar proteins, e.g., myotilin, desmin, and αB-crystallin, can be found [58, 62]. These myofibrillar myopathies are characterized by protein accumulation, autophagic vacuoles, granulofilamentous, as well as other types of protein aggregates [32, 56, 60].

While there are currently no pharmacological treatments for patients with genetically determined myofibrillar myopathies [60], patients with inflammatory myopathies, like IMNM or overlap myositis, can be treated with various immunosuppressants, though IBM patients are usually refractory to treatment [38]. Compared to other non-IBM inflammatory myopathies, Ku + myositis patients are reported to be less likely treated with immunosuppressive treatment [16]; however, the authors did not describe, whether therapy was also less successful in Ku + myositis patients.

Our study aimed to explicitly explore clinical and myopathological aspects in patients with anti-Ku-autoantibodies and myositis that had undergone skeletal muscle biopsy. We used comparative analyses of morphology, immunohistochemistry, transcriptomic and proteomic studies, especially focusing on autophagy and promotors of cell stress to identify pathophysiological pathways in the spectrum of Ku + myositis patients.

## Patients and methods

### Patients and biopsy specimens

We investigated 37 patients that were labeled as potentially Ku + myositis. Therefore, we selected all available patients from 2003 on, who underwent muscle biopsy with suspicion of myositis and who were reported to have Ku + antibodies by the physician. These 37 biopsies were then analyzed for myositis and a structured diagnostic work-up was performed. Biopsies not showing signs of myositis were excluded. In all other cases, clinical data were retrieved and re-checked for anti-Ku-positivity as well as systematically analyzed. Taken together, from the original cohort of 37 patients, 11 patients were excluded as they did not present with histopathological features of myositis (six patients), as analysis of the reports did not unequivocally reveal anti-Ku-positivity (no Ku-antibody in two cases, low anti-Ku-titer in EUROLINE blot and/or stronger concomitant MSA/MAA in two other cases) or as overall findings were not conclusive (one patient with IBM). In the end, clinical data of 26 patients with clinical and morphological myositis and positive anti-Ku-autoantibody status, tested by EUROIMMUN lineblot [40] from 5 German and 2 French centers, were included.

Skeletal muscle biopsy specimens from 12 IMNM patients and 7 IBM patients fulfilling diagnostic criteria [2, 36] and eight non-disease controls (NDC) were used in comparative analyses. NDC biopsies were obtained from patients that underwent biopsy for diagnostic purposes, and who did not show any alterations in muscle biopsies in the routine and extended diagnostic panel, and did not show any laboratory abnormalities.

Written informed consent was obtained from all patients and the Charité ethics committee granted ethical approval (EA2/163/17, AZ 07/09).

### Information on methods

All muscle biopsy specimens have been cryopreserved immediately after removal at -80°C prior to diagnostic work-up.

#### Morphologic analysis

Diagnostic conventional histology and enzyme histochemistry reactions (H&E, Gömöri trichrome, COXSDH, non-specific esterase, alkaline phosphatase, ATPases 4.3, 4.6, 9.4, Elastica van Gieson, MHC-class I, MHC-class II, CD3, CD8, CD31, CD45, CD68, C5b-9, CD20, CD138, Siglec/CD169, MHC-neonatal, MHC-developmental, ISG15, MxA, p62, LC3, Myotilin, Desmin, BAG3, HSP70) were performed on 8 µm-thick cryostat sections according to international recommendation [18] and as described previously [50]. To ensure negative staining results, we studied so-called irrelevant antibodies with the same antibody panel for validation. Additionally, where necessary, appropriate positive and negative controls were used and a physiological control or a normal muscle was used as negative control for all reactions as referenced [63]. Double immunofluorescence stains with p62, Myotilin, HSP70, Desmin, αB-Crystallin, and beta5i were performed as follows: slides were blocked with goat serum for 30min at room temperature, followed by simultaneous application of the primary antibodies (information see below), overnight at 4°C. After washing for 2 × 5min in PBS corresponding secondary antibodies (goat anti-mouse AF488, goat anti-rabbit AF488, goat anti-mouse Cy3, or goat anti-rabbit Cy3, 1:100) were incubated for 1 h at room temperature. After a final washing step (2 × 5min), slides were mounted with 4ʹ, 6-diamidino-2-phenylindole (DAPI) containing medium and stored at 4 °C. The antibodies used for morphological analysis are listed in Supplementary Table 2.

#### Semi-quantitative and qualitative analysis

A semi-quantitative analysis of inflammation and necrosis was performed: 0 negative/not present, 1 mildly positive/present, 2 moderately positive/present, and 3 strongly positive/present. This semi-quantitative analysis was performed for the following features: necrosis, vacuoles, endomysial infiltration, regeneration, MHC-class I, and MHC-class II [50, 52].

A qualitative analysis was performed with 0 negative/not present and 1 positive/present for: eosinophils, enlarged or thickened capillaries, myopathic features, myophagocytosis, neurogenic features, myofiber grouping, presence of (above age-related average) COX negative-SDH positive fibers, and perimysial alkaline phosphatase.

A qualitative descriptive analysis was performed for C5b-9 (negative, aggregates, sarcolemmal lining, and positive on capillaries), p62/LC3/myotilin (negative, capping, sarcoplasmatic aggregates, non-specific sarcoplasmatic faint positive), ISG15 (negative, on macrophages, on myofibers), MxA (negative, on sarcoplasm, on macrophages), and CD31 (negative, enlarged vessels, loss of capillaries).

A quantitative analysis was performed for leucocytic infiltrates with counting of 10 high power fields (HPFs), based on the microscope used and the respective oculars (Olympus WH10x-H/22) ≙ 0.16 mm^2^) for: CD45, CD8, CD68, CD20, CD138, CD169/Siglec1, and CD3.

Initially, the biopsies were analyzed and diagnosed without consideration of the patients’ serostatus as part of the standard clinical work-up. When the study was designed, the biopsies were selected based on their anti-Ku positivity. The evaluation and scoring was performed by three myopathologists (WS, HHG, and AU) and one rheumatologist (MTH) without knowledge of other clinical or laboratory parameters like, for example, CK values or organ involvement.

#### Electron microscopy

Muscle tissues were fixed with 2.5% glutaraldehyde in 0.1 M sodium cacodylate buffer, postfixed with 1% osmium tetroxide in 0.05 M sodium cacodylate, and dehydrated and embedded in Renlam resin. Uranyl acetate and phosphotungstic acid were used for contrasting. 70-nm ultrathin sections were cut using an ultramicrotome and an Ultra 35° diamond knife (Diatome), stretched with xylene vapor, collected onto pioloform-coated slot grids, and then stained with lead citrate. Standard transmission electron microscopy was performed using a Zeiss 906 microscope in conjunction with a 2k CCD camera (TRS).

#### RNA extraction and quantitative reverse transcription PCR (qRT-PCR)

Total RNA was extracted from whole muscle specimens using Trizol/chloroform extraction, followed by cDNA transcription using the High-Capacity cDNA Archive Kit (Applied Biosystems, Foster City, CA). For qPCR reactions, 10ng cDNA was used for subsequent analysis, using an Applied Biosystems™ QuantStudio™ 6 Flex Real-Time PCR System (ThermoFischer, Waltham, MA; USA, running conditions: 95°C 0:20, 95°C 0:01, 60°C 0:20, 40 cycles). Targeted transcripts were run as triplicates and the reference gene *PGK1* has been included as internal control to normalize the relative expression of the targeted transcripts. The TaqMan® Gene Exp Assay (Life Technologies/ThermoFisher) are as follows:

αB-Crystallin/*CRYAB* Hs00157107_m1, *CCL5* Hs00982282_m1, *CD68* Hs02836816_g1, *CD169/SIGLEC1* Hs00224991_m1, *LC3/MAP1* Hs01076567_g1, *LMP2/PSMB9* Hs00160610_m1, *LMP7/PSMB8* Hs00544760_g1, *PSME1* Hs00389210_g1, *PSME2* Hs01923165_u1, *p62/SQSTM* Hs01061917_g1, *PGK1* Hs99999906_m1.

Gene expression is illustrated by the logarithmic fold-change (RQ = 2^–ddCt^) values between patient´s and non-disease controls (NDC). For gene transcripts that were not expressed in NDCs, dCt-values are shown.

#### Unbiased proteomic profiling

Proteomic profiling on muscle protein extracts and subsequent data analysis were carried out as described before [25]. In short, muscle tissue of Ku + patients were lysed in 200 μl of 50 mM Tris–HCl (pH 7.8) buffer, 5% SDS and complete ULTRA protease inhibitor using a Bioruptor® sonication device at 4 °C. To ensure complete lysis, an additional sonication step was performed by ultrasound followed by centrifugation. Protein concentration of the supernatant was determined by BCA assay according to the manufacturer's protocol. Disulfide bonds were reduced by addition of Tris(2-carboxyethyl)phosphine hydrochloride (TCEP), and free sulfhydryl bonds were alkylated with.

From each sample, 100 μg of protein was used for proteolysis utilizing the S-trap protocol (Protifi) and a protein-to-trypsin ratio of 20:1. Proteolysis was stopped by acidification of the sample with formic acid.

All proteolytic digests were checked for complete digestion after desalting by monolithic column separation on an inert Ultimate 3000 HPLC. A binary gradient was applied. UV traces were recorded at 214 nm.

The proteomic signature of muscle biopsy specimens derived from Ku + myositis patients was compared to the protein status in NDC muscle biopsies. Ku + patients’ biopsies were studied in two groups, namely, severely and mildly inflammatory Ku + cases. This dichotomy was chosen based on the clear distinction of aggregates between these patients’ sub-cohorts: mildly inflammatory biopsies showed only a few fibers with aggregates, and these were mainly found in the center of the sarcoplasm, while severely inflammatory biopsy specimens additionally showed cap-like aggregates underneath the sarcolemma. Additionally, evaluation of the most upregulated proteins was also performed in IBM and IMNM samples.

#### Statistical analysis

Since this is an exploratory and descriptive study, sample sizes are not based on a priori power calculation, but based on previous studies. Statistic tests are only used for description, not test statistic purposes.

Data are presented as violin plots. Quantitative variables of mRNA transcripts were analyzed by Mann–Whitney *U* test or Kruskal–Wallis test followed by Bonferroni–Dunn correction for multiple comparison. The level of significance was set at *p* < 0.05. GraphPad Prism 9.02 software (GraphPad Software, Inc., La Jolla, CA, USA) was used for statistical analysis and visualization.

## Results

### Ku + myositis occurs predominantly in female patients either as isolated myositis or as systemic-sclerosis overlap

The 26 biopsy specimens of patients with Ku + myositis entailed biopsies from France and Germany (detailed information on biopsy results and patients’ characteristics in Supplemental Material 1, 2 and 3).

92.3% of the patients were female with a mean age of 53 years (median 56.5 years, range 20–78 years) at the time of biopsy. The disease duration until a muscle biopsy was performed was, on average, 18.2 months. The patients primarily presented with isolated myositis (30.8%, non-specified myositis, ‘poly’ or necrotizing myositis) or overlap with systemic sclerosis (SSc, 30.8%). Furthermore, overlaps with rheumatoid arthritis (19.2%), Sjögren’s syndrome (11.5%), and systemic lupus erythematosus (SLE, 11.5%) were reported (Table 1).

**Table 1:** Clinical data of patients with anti-Ku autoantibodies

*n* = 26	
Age in years mean (min–max)	53 (20–78)
Female *n* (%)	24 (92.3)
Disease duration in months mean (SD, min–max)	18.2 (18.5, 2–60)
Rheumatological diagnosis n (%)	
Isolated myositis (non-specified myositis or ‘Poly’, necrotizing myositis)	8 (30.8)
Overlap systemic sclerosis	8 (30.8)
Overlap rheumatoid arthritis	5 (19.2)
Overlap Sjögren’s syndrome	3 (11.5)
Overlap systemic lupus erythematosus	3 (11.5)
Not reported	2 (7.7)
ANA positivity *n* (%*)	22 (88.0)
Isolated Ku-antibody positivity *n* (%*)	11 (44.0)
CK elevation present *n* (%*)	23 (92.0)
CK elevation mean (SD, min–max) U/l (< 145U/l standard)	1772 (1639, 218–7355)

All patients presented with myalgia, and elevation of creatine kinase (CK) levels. Regarding extra-muscular symptoms, arthralgia, interstitial lung disease (ILD), pulmonary arterial hypertension (PAH), and cardiac involvement were the main features (see Table 2)

*n* = number of patients

*% of reported cases

All patients reported generalized myalgia, and 23/25 (92.0%) showed creatine kinase (CK) elevation. Proximal muscle weakness was described in 12/22 patients (54.5%) and dysphagia in 10/17 patients (58.8%). Regarding extra-muscular symptoms, arthralgia was reported by 16/20 (80.0%) of the patients, interstitial lung disease (ILD) in 8/21 patients (38.1%), pulmonary arterial hypertension in 6/18 patients (33.3%), and cardiac involvement (mostly myocarditis, but also non-specified cardiac involvement with, e.g., arrhythmia or reduced ejection fraction) in 14/21 patients (61.9%) (Table 2).

**Table 2:** Subgroup analysis of clinical data comparing Ku + myositis patients with isolated myositis (includes ‘poly’myositis and necrotizing myopathy), overlap with systemic sclerosis (SSc) as well as with systemic lupus erythematosus (SLE)

% (*n*/reported)	All (*n* = 26)	Overlap SSc (*n* = 8)	Overlap SLE (*n* = 3)	Isolated myositis (*n* = 8)
Muscle weakness	54.5% (12/22)	66.7% (4/6)	0% (0/1)	37.5% (3/8)
Arthralgia	80.0% (16/20)	75.0% (6/8)	100% (1/1)	66.7% (4/6)
Raynaud's phenomenon	63.6% (14/22)	87.5% (7/8)	50% (1/2)	42.9% (3/7)
Sclerodactyly'	38.1% (8/21)	87.5% (7/8)	0% (0/2)	0% (0/6)
Dermatomyositis-like skin findings	33.3% (8/24)	62.5% (5/8)	100% (2/2)	14.3% (1/7)
Dysphagia	58.8% (10/17)	83.3% (5/6)	0% (0/1)	60% (3/5)
ILD	38.1% (8/21)	57.1% (4/7)	100% (1/1)	28.6% (2/7)
PAH	33.3% (6/18)	42.9% (3/7)	50% (1/2)	16.7% (1/6)
Renal involvement	23.1% (3/13)	25.0% (1/4)	100% (2/2)	0% (0/4)
Cardiac involvement	61.9% (14/21)	50.0% (4/8)	50% (1/2)	83.3% (5/6)
Neuropathy	52.6% (10/19)	40% (2/5)	0% (0/1)	42.5% (3/7)
Creatine kinase mean (< 145U/l standard range)	1772 U/l	1119 U/l	1887 U/l	2123 U/l

Patients presented more frequently dysphagia, Raynaud’s phenomenon, and sclerodactyly in the systemic sclerosis group. Regarding organ involvement, ILD and renal involvement were more common in systemic sclerosis overlap and systemic lupus erythematosus overlap, whereas cardiac involvement was more present in isolated myositis

Subgroup analysis comparing isolated myositis and overlap with systemic sclerosis or with systemic lupus erythematosus showed that patients presented more frequently dysphagia, Raynaud phenomenon and sclerodactyly in the systemic sclerosis group (SSc vs. isolated myositis/SLE overlap: 83.3% vs. 60%/0%, 87.5% vs. 42.9%/50%, and 87.5% vs. 0%/0%, respectively). Regarding organ involvement, ILD and renal involvement were more common in systemic-sclerosis overlap and systemic lupus erythematosus overlap (50.0% and 100% vs. 28.6% and 25% and 100% vs. 0%, respectively), whereas cardiac involvement was more present in isolated myositis (83.3 vs. 50.0% in SSc and SLE) (Table 2). Additional comparison of subgroups with isolated Ku-antibodies and concomitant antibodies regarding clinical aspects is displayed in Supplementary Table 3.

### Histopathology findings show combined features of myofiber necrosis, myophagocytosis, endomysial inflammation, and vacuoles

In the qualitative and semi-quantitative histopathological analysis of Ku + myositis, we identified a wide spectrum of myositis, from mild to very severe features. All biopsies displayed variable myopathic features (Supplemental Material 3). As a common pattern, myofiber necrosis in 88.5% of the biopsies, endomysial inflammation in 84.6%, myophagocytosis in 73.1%, and vacuoles in 60% were detected. Only 4 specimens (15.4%) showed scattered COX negative-SDH positive fibers, below age-related numbers [21]. Furthermore, many biopsies showed thickened or enlarged capillaries in the endomysium (21/25, 84.0%) with ultrastructural abnormalities comprising multi-lamellated basement membranes, increased numbers of pericytes intermingling with the thickened basement membranes (Fig. 4b). Interestingly, in 6 of 25 samples (24.0%), some eosinophils in the endomysium were identified additionally.

Sarcolemmal staining of myofibers was found in all biopsies for MHC-class I and in 69.2% of the biopsies for MHC-class II with a diffuse distribution and without perifascicular pattern. The inflammatory infiltrates were located in the endomysium and presented in a diffuse manner and with a focal enhancement around necrotic myofibers. These infiltrates consisted mainly of CD68 positive cells (mean of 210 cells/10 HPF = high power field ≙ 0.16 mm^2^), followed by CD45 positive cells (mean of 101 cells/10 HPF) and CD3 positive cells (76 cells/10 HPF), and CD8 positive cells (mean of 47 cells/10 HPF). B cells and plasma cells were scarce with CD20 positive cells (7 cells/10 HPF) and CD138 positive cells (12 cells/10 HPF), whereas follicle-like accumulations of lymphocytes were absent. CD169 positive macrophages (51 cells/10 HPF) were occasionally present in the inflammatory infiltrates (Supplementary Figs. 1 and 6). Complement deposition of C5b-9 showed sarcolemmal positivity in 17 of 25 biopsy samples (68%), whereas deposition on capillaries could be found in 7 muscle biopsies (28%). Of note, complement decorated the sarcoplasmic aggregates in 2 biopsy samples (8%).

Histomorphologically, isolated myositis biopsies showed more pronounced endomysial infiltration as well as MHC-class II expression compared to biopsies with SLE or SSc overlap (Supplementary Fig. 3). There was no significant difference between patients with isolated Ku-antibodies and concomitant antibodies (Supplementary Fig. 4).

On the gene expression level, we identified significant increase of *CCL5* in comparison to NDC and IMNM patients’ muscle samples; furthermore, *CD68* and *CD169* gene expression was upregulated compared to that in NDC muscle tissues (Supplementary Fig. 1).

**Fig. 1:**
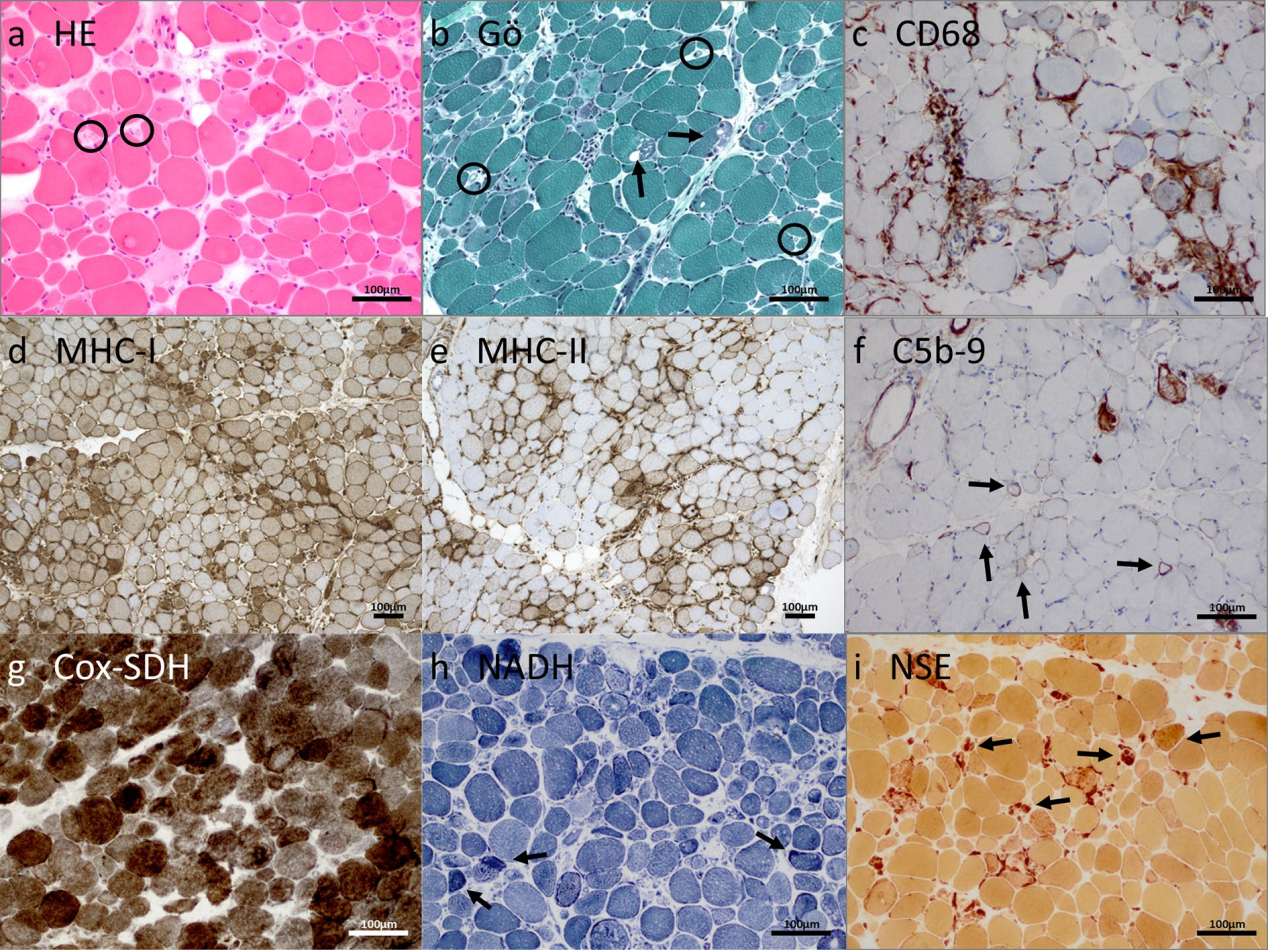
Histopathological and immunohistochemical stains of a representative Ku + myositis specimen. Histopathology of Ku + myositis showed a spectrum of mild to very severe myositis with all biopsies showing myopathic features including fiber size variation, rounded fibers and internalized myonuclei, furthermore, commonly necrotic myofibers, as well as myophagocytosis and presence of vacuoles (black arrow in **b**). Many biopsies showed thickened or enlarged capillaries in the endomysium (black circles, see also Fig. 4) (**a**, **b**). The inflammatory infiltrates were located in the endomysium, again diffuse and with a focal enhancement around necrotic myofibers, mainly consisting of CD68 positive cells (**c**) (for other inflammatory cells, please see Supplementary Fig. 5). All muscle biopsies stained with MHC-class I on the sarcolemma of the myofibers (**d**), and the majority of samples additionally stained with MHC-class II on the sarcolemma with patchy distribution (**e**). Complement deposition with C5b-9 showed decoration on the sarcolemma in the majority of biopsies (black arrows) (**f**). No COX negative-SDH positive fibers above age-related numbers were detected (**g**). Some biopsies showed subsarcolemmal dark aggregates representing areas of pathologic mitochondrial accumulation (**h**). Staining with non-specific esterase showed frequent myophagocytosis in most biopsies (**i**)

In general, the above-mentioned histopathological features of Ku + myositis may resemble IMNM (necrotic fibers) and IBM (inflammation and vacuoles). When comparing histopathological and immunohistochemical patterns observed in Ku + myositis, IMNM, and IBM skeletal muscle biopsy specimens, we found a more pronounced extent of necrotic fibers as well as inflammatory infiltrates in Ku + myositis than in IMNM. While necrotic fibers can occur in IBM, the extent of the lymphomonocytic infiltrates is even greater in IBM than in Ku + myositis. Furthermore, MHC-class II does not stain on myofibers in IMNM but is positive in most Ku samples. In contrast, MHC-class II stains most consistently and prominently on myofibers in IBM biopsies. We did not observe significantly elevated numbers of COX negative-SDH positive fibers in Ku + myositis (and not in IMNM), but they were detectable above age-related numbers in the muscle biopsy specimens derived from IBM patients. A representative biopsy sample with characteristic histopathological and immunohistochemical staining of Ku + myositis is provided in Fig. 1. Supplementary Figure 2 shows a comparison of representative biopsies from Ku + myositis, IBM, and IMNM. Supplementary Fig. 5 additionally shows a comparison of representative Ku + biopsies to dermatomyositis and antisynthetase syndrome.

### Autophagy, proteasome, and cell stress pathways are characteristically activated in Ku + myositis

**Fig. 2:**
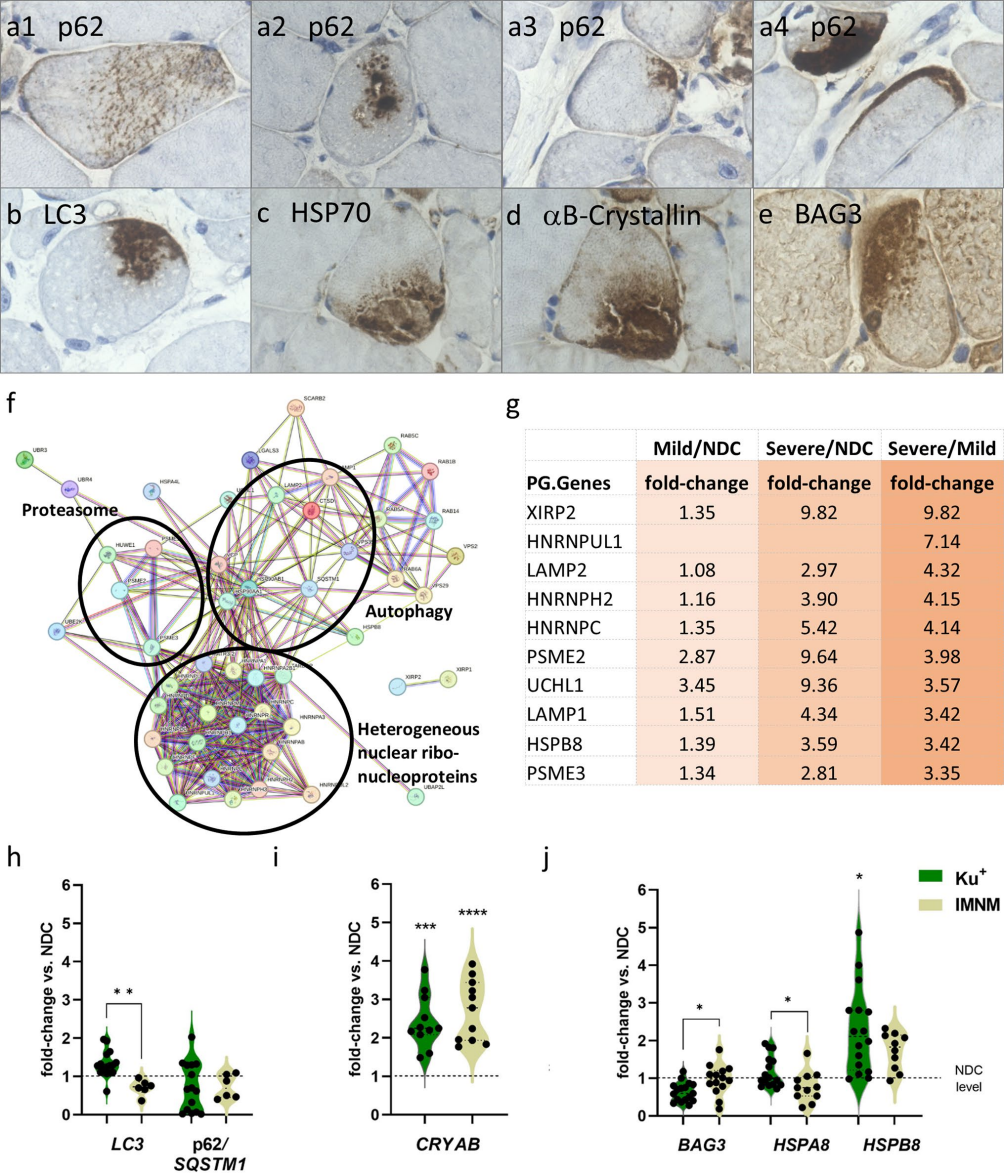
Autophagy pathways are activated in Ku + myositis on both gene and protein levels. Immunohistological staining of autophagy markers p62 revealed large aggregates in the sarcoplasm of non-necrotic myofibers, with aggregates located in the center of the sarcoplasm or appearing subsarcolemmally as large caps (**a1–4**). The larger aggregates were also positive for BAG3, αB-Crystallin and HSP70 (**b**–**e**). With an unbiased proteomic approach we identified multiple proteins being involved in regulation of autophagy (see Supplementary Table 1). As demonstrated by the STRING graph, these proteins form a dense interacting network (**f**). Of note, when comparing Ku + myositis to NDC we identified upregulated proteins, which, when comparing mild versus severe Ku + myositis patients, showed even higher extent in the development of the disease, as shown for the top 10 regulated autophagy and cell stress proteins (**g**) (for comparison of these proteins in IBM and IMNM, see Supplementary Table 4). Of note, three of the highest expressed proteins are part of the hnRNP machinery acting as key proteins in the cellular nucleic acid metabolism. Interestingly, in the analysis of autophagy genes, we could not find differences of p62/*SQSTM1* expression between Ku + myositis, NDC, and IMNM, but a significantly higher *LC3* expression in Ku + samples compared to IMNM samples (h). *CRYAB* was significantly more expressed in Ku + myositis and IMNM compared than in NDC (**i**), as was *HSPB8* for Ku + patients, while other chaperons (*BAG3*, *HSPA8*) were not changed compared to controls. However, a difference was seen between Ku + patients and IMNM, overall highlighting that not the quantity of gene expression but their functional implications (which seem variable) is more relevant in Ku + myositis compared to the other entities studied (**j**)

Of note, the autophagy markers p62 and LC3 revealed characteristic aggregates in the sarcoplasm of myofibers in 17/23 (73.9%) and 16/22 (72.7%), respectively, independent of vacuoles (Fig. 2). Myofibers containing aggregates of variable appearance were found in different quantities, positively correlating with the density of inflammatory infiltrates. Those aggregates were either located in the center of the sarcoplasm and often occupying a rather large area or appeared subsarcolemmally as variably sized caps (see Fig. 2 a1–4). Morphologically, these aggregates were different from the fine punctate p62 pattern described in IMNM [24] and the coarse p62 pattern in IBM muscle fibers [33, 47]. The large aggregates were also positive for the chaperones BAG3, αB-crystallin, and HSP70 (Fig. 2c–e).

To further elucidate the pathobiochemistry of Ku + myositis, especially regarding the altered protein clearance via autophagy, we next performed unbiased proteomics using a data-independent acquisition (DIA) approach. Unbiased proteomic profiling enabled the quantification of 3091 (2225 with two unique peptides) proteins across three different analytical sets (NDC, mildly inflammatory as well as severely inflammatory Ku + myositis muscle specimens, respectively, defined as severe Ku + and mild Ku + below). Of these, 180 proteins were differentially regulated between severe Ku + myositis and NDC, whereas 501 were dysregulated when comparing the mildly versus the severely inflammatory muscle biopsies, as some proteins were not found in NDC at all. By taking the latter data set as a basis, we next filtered the results for statistically significant dysregulated proteins being involved in the regulation of autophagy and proteasome functions and identified 48 proteins with increased abundance and compared the fold of change with the abundances of the respective proteins (Supplementary Table 1). As demonstrated by the STRING graph, these proteins form a close interactive network with three main functional clusters, which include (i) proteasome-related, (ii) autophagy-related, and (iii) heterogeneous nuclear ribonucleoproteins with the latter ones representing the most prominent class (Fig. 2f, g). Certain autophagy-related proteins were only found in Ku + patients and not in NDC ones, indicating their characteristic role in pathogenesis of Ku + myositis (Supplementary Table 1). As additional comparison, we included IMNM and IBM analyses (Supplementary Table 4). From the top 10 upregulated proteins in Ku + myositis, only few were found in IMNM (XIRP2, HNRNPH2, and PSME2), while the others were not detected. Interestingly, XIRP2 and PSME2 were upregulated by tenfold and eightfold similarly to Ku + Myositis. In IBM proteomics, none of the proteins were significantly altered and three of them not expressed at all.

In the analysis of transcripts encoded by autophagy-related genes, we did not find differences in *p62*/*SQSTM1* expression between Ku + myositis-, NDC- and IMNM muscles, but a significantly higher *LC3* expression in Ku + samples compared to IMNM samples. *CRYAB*, coding for αB-crystallin, on the other hand, was significantly more expressed in Ku + myositis muscles than in NDC muscles (Fig. 2h, i). Transcripts of *BAG3* and *HSPA8* (encoding for other chaperones), were significantly different between Ku + patients’ and IMNM muscle samples but not significantly altered when compared to NDC samples, while *HSPB8* was significantly increased in Ku + patients’ muscles (Fig. 2j). Between mildly and severely affected Ku + cases, no difference in the gene expression levels was seen (data not shown), overall highlighting that the variable functional implications of autophagy are likely more relevant rather than the quantity of gene expression in Ku + myositis compared to the other entities studied.

**Fig. 3:**
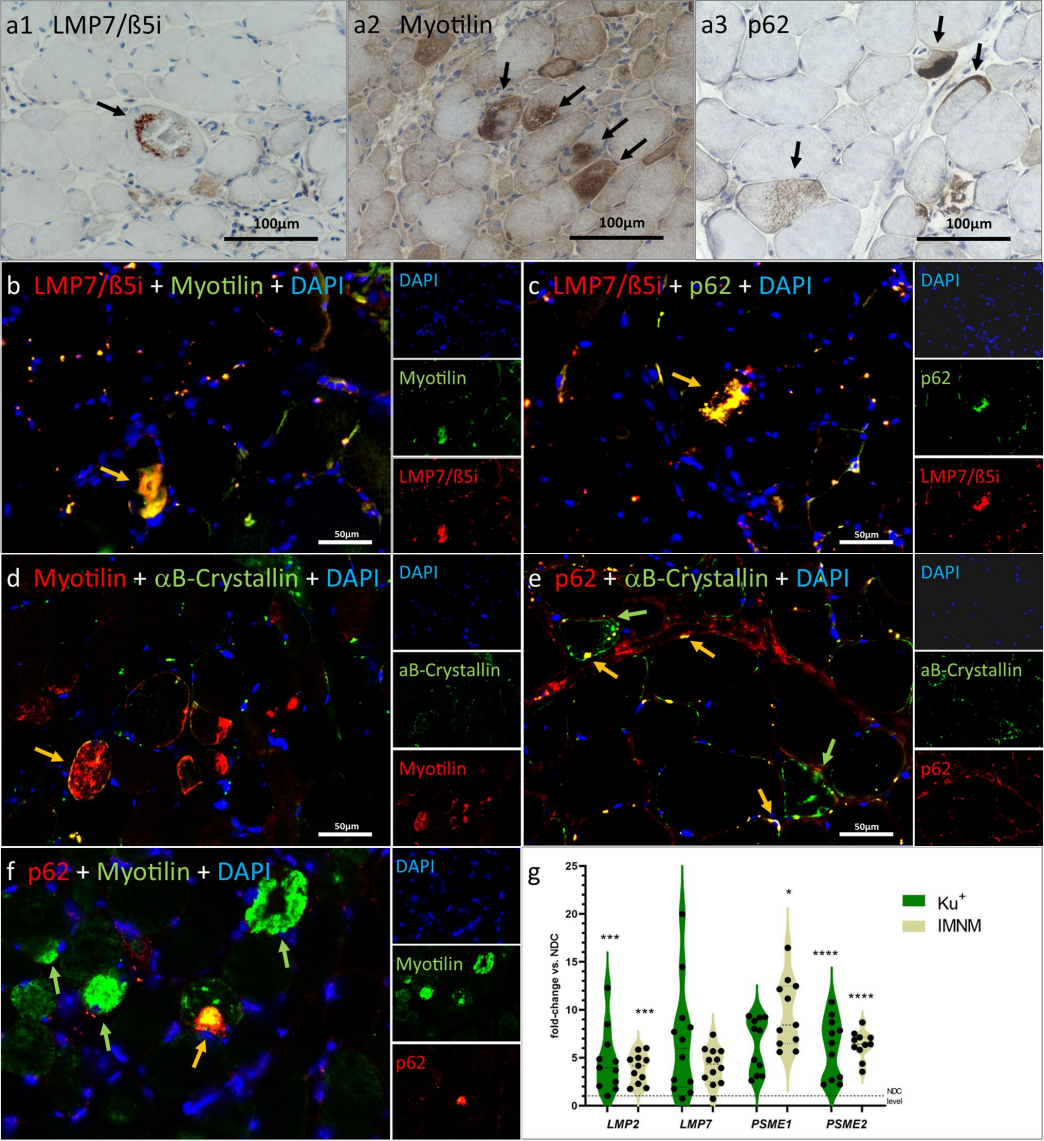
Aspects of pathological protein aggregation in Ku + myositis. Large sarcoplasmic and subsarcolemmal aggregates stain positive with the immunoproteasome molecule LMP7/β5i (black arrow, **a1**), the structural protein myotilin (as well as the intermediate filament desmin; not shown) delineates aggregates of dense staining (black arrows, **a2**), while most of the non-affected myofibers show a fine physiological positivity, and the autophagy molecule p62 (as well as LC3; not shown) highlights sarcoplasmic aggregates with different quality of the staining pattern as well (black arrows, a3). Myotilin-positive aggregates (AF488; green) co-localize with the immunoproteasome marker LMP7/β5i (Cy3; red) (**b**). The autophagy marker p62-positive aggregates (AF488; green) co-localizes with the immunoproteasome marker LMP7/β5i (Cy3; red) (**c**). And additional stainings show co-localization of myotilin stained fibers (Cy3; red) with the chaperone αb-Crystallin (AF488; green) (**d**), as well as small aggregates that are p62 + (Cy3; red) and αB-Crystallin + (AF488; green) (**e**). Myotilin-positive aggregates (AF488; green) also co-localize with the autophagy marker p62 (Cy3; red) (**f**). On the gene expression level, proteosomal markers (*LMP2*, *PSME2*) are significantly increased in Ku + patients versus non-diseased controls, while there is no significant difference compared to IMNM patients (**g**). Yellow arrows = double staining of both markers; green arrows = single staining of respective marker

Proteomic profiling additionally unraveled a profound up-regulation of proteasome factors, including proteins crucial for the composition of the immune proteasome (subunits LMP2, LMP7), which is specifically adapted for a role in MHC-class I antigen processing and CD8 + T-cell activation [71]. By immunohistochemical validation, we identified ß5i/LMP7 immunoreactivity appearing as sarcolemmal aggregation or subsarcolemmal caps, also with co-localization to autophagy proteins such as p62 (Fig. 3 a, c). In addition, on the gene transcript level, we identified significantly elevated gene expression of the immune proteasome markers *LMP2* and *LMP7*, and also of *PSME2* in Ku + biopsies in comparison to NDC muscle tissues (Fig. 3g).

### Ku + patient derived skeletal muscles feature protein aggregation

The cytoskeletal protein myotilin (in 17/20, 85% of the biopsies) and myofibrillar desmin showed aggregation in the sarcoplasm or subsarcolemmal capping of Ku + myofibers (Fig. 3a). Immunofluorescence-based staining studies showed co-localization of autophagy, immune proteasome, chaperone, and myofibrillar proteins within these protein aggregates (Fig. 3b–e).

**Fig. 4:**
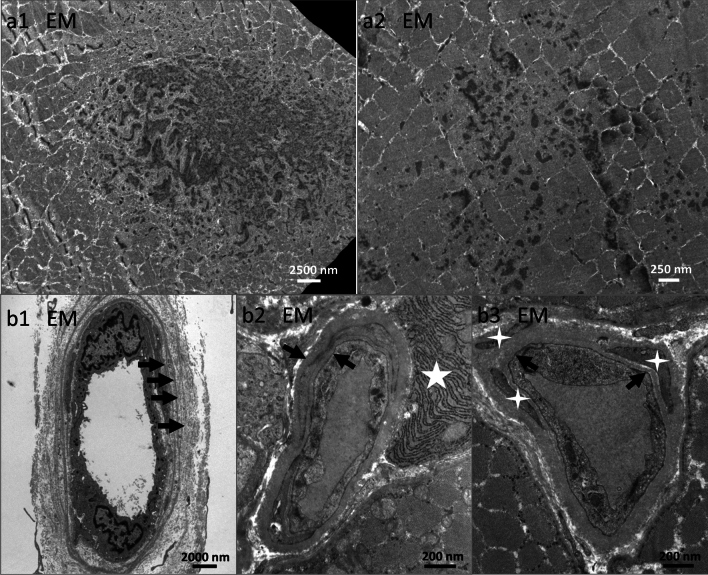
Ultrastructural analysis reveals disturbed contractile elements and changes in capillary basement membranes. Ultrastructural analysis highlights focally aggregated and disrupted contractile elements which is very pronounced in (**a1**) and less pronounced probably in *statu nascendi* in (**a2**). Those alterations are very similar to the ones detectable in genetic protein-aggregate myopathies. The capillaries frequently showed multilayered basement membranes exceeding 4 layers (black arrows, **b1**), and basement membranes may also appear more homogeneously thickened (black arrows, **b2**); note the prominently activated rough endoplasmic reticulum (white star, **b2**). Pericytes (white small stars) are intermingling with thickened basement membranes (black arrows, **b3**).

Analysis by electron microscopy (EM) demonstrated disruption of the contractile apparatus with Z-band disruption and accumulated intermyofibrillar filamentous material very similar to what is known from genetic myofibrillar myopathies, e.g., autosomal dominant desmin mutations. These cytoskeletal perturbations were not identified in IMNM and in IBM specimens by EM (Fig. 4a). We did not identify tubulofilamentous inclusions in perinuclear or vacuolar or intranuclear localizations that are characteristic of IBM. However, we did see occasional tubuloreticular inclusions that are characteristic of interferon-mediated endothelial damage occurring in lupus erythematosus-associated Ku + myositis muscles (Supplementary Fig. 7).

## Discussion

Our study clarifies the placement of Ku + myositis in the wide spectrum overlap myositis, especially associated with scleromyositis. Our initial cohort contained some patients, that did not show signs of myositis despite a positive Ku-antibody test, and that were therefore excluded from our analysis. Most of the patients had isolated myositis or overlap with systemic sclerosis (30.8% respectively), whereas overlap with SLE and Sjögren’s syndrome was present in 11.5%. It is known that the presence of Ku-antibodies is not necessarily associated with myositis but can also occur in healthy individuals or with other organ involvement than myositis [64]. Interestingly, there seem to be regional differences, as in the United States, Ku-antibodies are found in SLE, whereas, in Japan, they are often associated with myositis or systemic sclerosis [17, 48].

In our cohort, cardiac involvement was seen in nearly 2/3 of the patients, predominantly occurring in patients with isolated myositis. Myocarditis in Ku + myositis is also a commonly described feature: one study meta-analyzed a prevalence of 23% [66], whereas another one reported on cardiac involvement in four out of four Ku + SSc patients [15]. These variances may indicate the problem of differently interpreted or reported cardiac involvement on the one hand or underdiagnosed cardiac involvement in some other cohorts. In our cohort, SSc and SLE patients with slightly lower CK levels had more frequently concomitant ILD than the isolated myositis patients with higher CK levels. Overall, ILD occurred in 38.1% of all included patients. Contrarily, Spielmann et al. detected elevated CK levels as risk for ILD [65], and Rigolet et al. associated Ku-antibodies with concomitant myositis with higher ILD frequency than in Ku + patients without myositis [55].

In our current study, we performed an *in-depth* morphological analysis and transcriptional and proteomic analysis of biopsies from 26 Ku + patients with associated myositis. We could define a distinct pattern in Ku + myositis with diffuse and focally enhanced sarcolemmal MHC-class I expression, endomysial macrophages-predominant lymphomonocytic inflammation, myophagocytosis and, in contrast to IMNM [47], with concomitant MHC-class II expression, as well as characteristic protein aggregates and vacuoles. Previous studies show partly contradictory results regarding the histopathological aspects, reporting a pattern of combined necrosis, inflammation, and MHC-class I positivity on myofibers [55, 68], general inflammatory aspects or CD68 + predominant infiltration [23, 72], or necrosis without any inflammation [39]. Often, the pathological aspects were described as ‘IMNM-like’ [67, 72] according to the ENMC criteria for IMNM [2]. Our results indicate that the combined pattern of MHC-class I and II expression, the endomysial inflammation, as well as the distinct protein aggregation and vacuoles represent a ‘well-recognizable’ morphological picture that enables differentiation of Ku + myositis from other IIMs.

Furthermore, placing Ku + myositis in the concept of overlap myositis and scleromyositis [41], we could identify typical SSc-related features in our biopsies. 81% of our biopsies (not only SSc-associated biopsies) had enlarged and thickened capillaries. Ellezam et al. suggest that the capillary pathology with multilayered basement membranes is a central feature of scleromyositis histopathology [20]. Additionally, we could detect ultrastructural changes of the basement membranes with thickening and reduplication, characteristic features of the ‘minimal myositis with capillary pathology’ (MMCP) established in SSc patients [63]. Moreover, we identified homogenous thickening of the basement membrane resembling the previously described pipestem capillaries in necrotizing myopathy biopsies [59]. As some scleromyositis patients with SSc-overlap antibodies (like Ku-antibodies) may lack typical features of skin involvement like sclerodactyly [34], it might be possible that patients with isolated myositis and aspects of capillary pathology as well as necrotizing myopathy may develop specific clinical SSc features later on.

Histomorphologically, the Ku + biopsies of patients described as SLE overlap in our cohort presented with the same pattern as the other biopsies, including enlarged and thickened capillaries, but they featured additional lupus-related tubuloreticular inclusions in endothelial cells. Myositis in SLE is rare and its pathological patterns show a wide range from dermatomyositis to necrotizing myopathy [12, 70]. Furthermore, in presence of Ku-antibodies, SLE-myositis patients often exhibit scleroderma features as well [12]. Nevertheless, to identify possible differences, our cohort of three patients with SLE-myositis overlap is too small to draw definite conclusions.

Beyond the placement of Ku + myositis among the inflammatory myopathies and in the cluster of scleromyositis, we could identify distinct sarcoplasmic and subsarcolemmal aggregates that were composed of disrupted myofibrillar filaments and Z-band structures. These aggregates have been identified similarly in many of the so-called protein-aggregate myopathies with genetic backgrounds, but not in myositis and are linked to altered autophagy, immunoproteasomal function, and specific mechanisms involved in cell stress:

Autophagy markers like p62 have been identified with a very explicit staining pattern in IMNM (fine granular sarcoplasmic) and IBM (focal dense vacuoles-related staining) [14, 24, 47], both diagnostically relevant and helping to understand pathophysiological pathways. In our study, the accumulation of autophagy markers like p62 and LC3 on sarcoplasmic aggregates and subsarcolemmal caps differed substantially from that in IMNM and IBM. Furthermore, ultrastructural examination disclosed that these sarcoplasmic aggregates correspond to filamentous deposits and disruption of the contractile apparatus, which is at stark variance with the ultrastructure of IMNM (fine autophagolysosomal compartments) [51] and IBM (extensive vacuolar debris and tubulofilaments) [46]. Similarly to the very specific accumulation of autophagy proteins, chaperones also showed either centrosarcoplasmic or cap-like aggregation in our Ku + biopsies, again in contrast to the fine granular pattern co-localizing with autophagy markers in IMNM and in the focal dense pattern confining to vacuoles in IBM [24]. Immunofluorescence staining showed co-localization of autophagy proteins with key chaperones. Furthermore, we identified aggregation and co-localization of the aggregates with the immune proteasome marker β5i as well as its increased gene expression (*LMP7*). The immune proteasome is responsible for protein degradation, but also for MHC-class I antigen presentation [45] and is involved in T-cell expansion and macrophage activation [6]. In rheumatoid arthritis and other autoimmune disease models, co-inhibition of immune proteasome subunits leads to amelioration of the diseases [30]. We have previously shown that the immune proteasome is involved in MHC-class I overexpression and the regulation of cytokines in myositis [11], therefore substantially contributing to pathophysiology.

Interestingly, BAG3 or αB-crystallin aggregation as well as aggregation of myofibrillar structural proteins like myotilin and desmin in the Ku + biopsies resemble aggregation in myofibrillar myopathies [62]. These myopathies can be caused by, e.g., *BAG3* mutations and lead to muscle weakness, cardiomyopathy, and neuropathy [61, 62]. Protein accumulation, autophagic vacuoles, and granulofilamentous material or cytoplasmic bodies can be detected in myofibrillar myopathies by electron microscopy [60].

To further analyze the observed protein aggregation in our cohort of Ku + myositis we performed proteomic analysis. Besides the up-regulation of autophagy and proteasome-related proteins, proteomic analysis identified an increase of many heterogeneous nuclear ribonucleoproteins (hnRNPs) compared to NDC. In our comparison cohorts of IBM and IMNM, only very few of these proteins were upregulated.

HnRNP proteins are involved in many aspects of nucleic acid metabolism, especially in the splicing and stabilization of messenger ribonuclein acids (mRNAs). They interact with DNA repair mechanisms and telomere function [26, 28]. Mutations of the *hnRNPA1* gene subtype are reported to cause a genetic form of IBM, with a chronic vacuolar myopathy with ultrastructural tubulofilamentous inclusions lacking inflammation [29] and hnRNPA1 aggregates co-localized with p62 in small aggregates as well [29]. On the one hand, variants of hnRNPs interact with the Ku protein in DNA-repair mechanism, and this interaction can be disturbed by cellular stress [28]. On the other hand, decreased expression of the Ku70-subunit is associated with decreased DNA repair and cellular senescence [31] and Ku is involved in telomere function [54]. Ku itself is part of the DNA–protein kinase complex, and can build a ribonucleoprotein complex together with HEXIM1, which is involved in the innate immune response, especially in type 1 interferon induction [44]. The DNA–protein kinase complex is necessary to phosphorylate, for example, hnRNP-U, to properly address DNA breaks [8]. Double string breaks (DSB) are known to result in aggregate formation at the locus of DNA damage (so-called ‘foci’) [9]. Additionally, Ku is an inhibitor of apoptosis via inhibition of the proapoptotic factor Bax [57], and reduction of Ku70 proteins led to accumulation of ubiquitylated Bax [3]. Bax activation, on the other hand, is reported to induce autophagy via LC3 [35] and Ku regulates the transcription of the *hsp70* gene [1]. Similar to its interaction with hnRNP, Ku interferes with various heat shock proteins leading to DSBs [19]. Interestingly, Pinal-Fernandez et al. could recently show, that autoantibodies in myositis can be internalized in muscle fibers and can lead to dysfunction of the respective autoantigen [49]. These data may suggest a possible interaction between Ku, Ku-antibodies, hnRNP-system, and autophagy/chaperone systems, resulting in disturbed DSB repair and protein aggregation. Moreover, the circumstance, that specific molecules, which have a pathogenic counterpart in genetic myopathies (like hnRNP [27] and BAG3 [62]), interact with an antigen and operate in the pathogenesis of myositis has been seen in IMNM as well: Here, anti-HMGCR autoantibodies recognize the HMG-CoA-reductase protein. Mutations in HMGCR were shown to cause recessive dystrophic myopathy with myofiber phagocytosis similar to what is known from IMNM pathomorphology as well as rimmed vacuoles and myofibrillar disruption on ultrastructural analysis [43]. This suggests that autoantibodies attacking HMGCR and pathogenic genetic variants of the gene encoding the same molecule could lead to very similar abnormalities in the affected muscle tissues.

To the best of our knowledge, this is the first study investigating a large cohort of Ku + myositis patients *in-depth* by morphological, transcriptional, and proteomic means in a retrospective manner. Nevertheless, a limitation of this research is, that the selection for primary screening of biopsies was performed with the EUROLINE blot. A probable high false-positive rate for Ku-antibodies in the EUROLINE blot was reported [16, 69]. As these studies showed false-positive results especially with low titer-antibodies, we tried to minimize the possibility of false positivity with excluding very low Ku-titer and concomitant stronger MSA/MAA. Additionally, three patients were stated to be anti-nuclear antibody (ANA) negative. False-negative reported ANA due to differences in laboratory performance especially by indirect immunofluorescence or differences in cut-off values [13] might be a possible explanation as retrospective evaluation did not allow confirmation of ANA or antibody results. Although the possibility of false-positive Ku-antibodies could not be entirely excluded, our histopathological analysis revealed consistent pattern across all biopsies. This pattern differs from those associated with other known idiopathic inflammatory myopathies (IIMs), suggesting the presence of a distinct type of myositis. Neuropathy was described in many patients, but it was not stated whether the neuropathy was part of the rheumatological disease or had another underlying cause. Additionally, clinical aspects that were not reported initially could not be retrospectively identified. Therefore, missing data were indicated as such in the analysis.

To conclude, this study indicates that Ku + myositis is a unique subtype of overlap myositis with a distinct histopathological pattern of myofiber necrosis, MHC-class I and II positivity, endomysial inflammation, and vacuoles, whereby protein aggregation is linked to altered autophagy, chaperone, and immunoproteasome dysfunction, distinct from those in IBM and IMNM. Myofibrillar protein aggregation as well as hnRNPs’ dysregulation identifies Ku + myositis as an acquired inflammatory protein-aggregate myopathy.

### Supplementary Information

Below is the link to the electronic supplementary material.

Supplementary file1 (DOCX 2784 KB)

## Data Availability

All important data are available in the manuscript or as supplemental material online. Other data as raw data are available upon reasonable request to the corresponding author.
