# Supplementary material for: Anti-Ku + myositis: an acquired inflammatory protein-aggregate myopathy
Source: Acta Neuropathol. 2024 Jul 16;148(1):6. doi: 10.1007/s00401-024-02765-3 (PMC11252205; doi:10.1007/s00401-024-02765-3)
Supplement: Supplementary file 1 — Supplementary file1 (DOCX 2784 KB) [file 401_2024_2765_MOESM1_ESM.docx]

**Anti-Ku+ myositis – an acquired inflammatory protein aggregate myopathy**

*Marie-Therese Holzer^1,2^, Akinori Uruha^2,3,4^, Andreas Roos^5,6,7^, Andreas Hentschel^8^, Anne Schänzer^9^, Joachim Weis^10^, Kristl G. Claeys^,11,12^, Benedikt Schoser^13^, Federica Montagnese^13^, Hans-Hilmar Goebel^2^, Melanie Huber^14^, Sarah Léonard-Louis^15^, Ina Kötter^1^, Natalie Streichenberger^16^, Laure Gallay^17^, Olivier Benveniste^18^, Udo Schneider^3^*, Corinna Preusse^2^*, Martin Krusche^1^* and Werner Stenzel^2^**


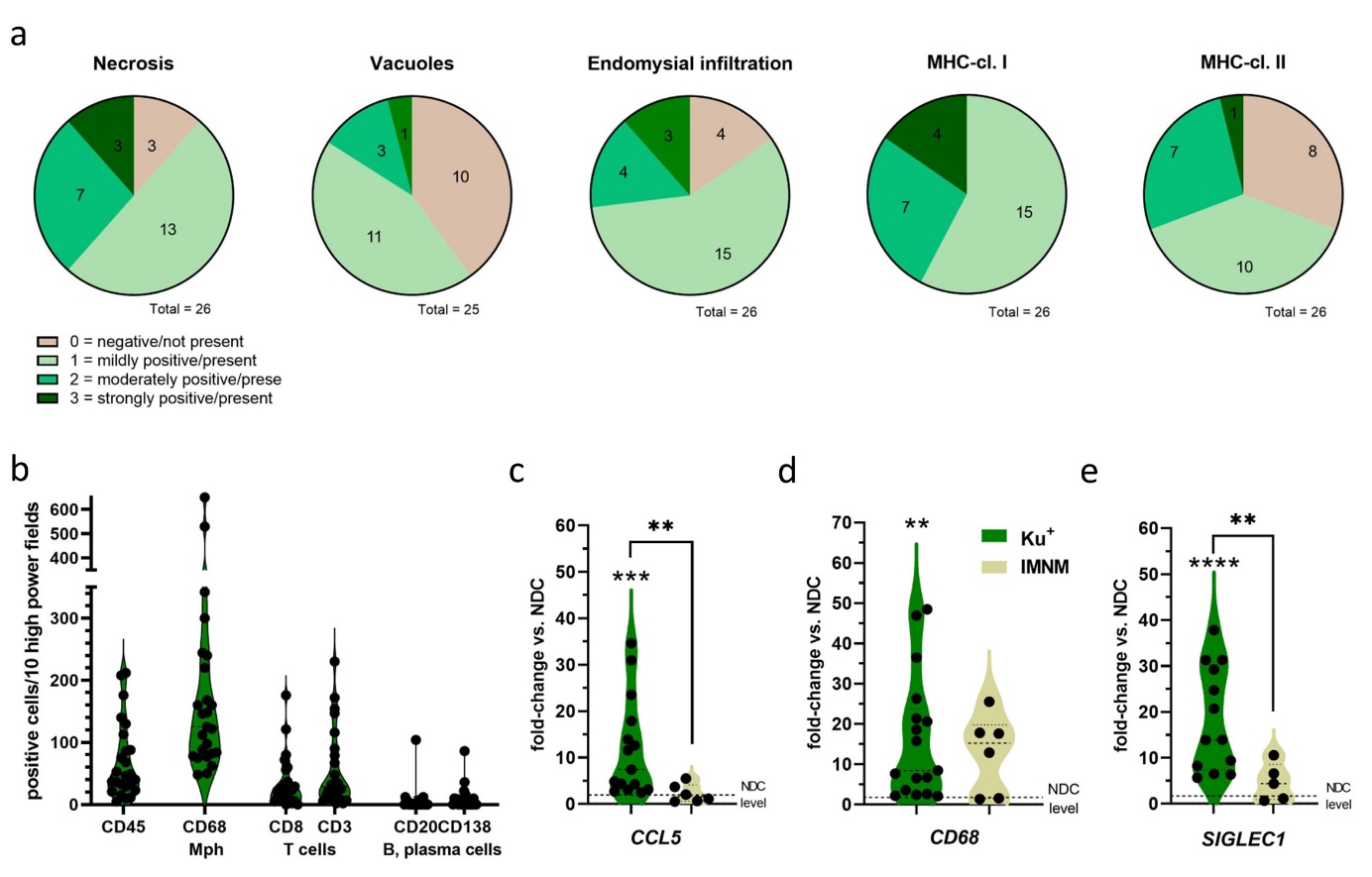


**Supplementary Fig. 1 Scoring of histomorphological aspects of Ku+ biopsies (a+b) and gene transcript analyses in Ku+ patients in comparison to immune-mediated necrotizing myopathy (IMNM) and non-disease controls (NDC) (c-e)**

a Scoring of characteristic histopathological findings in 26 Ku+ biopsies including necrosis, vacuoles, endomysial infiltration as well as MHC-class I and II positivity on myofibers in a semiquantitive manner. b Quantitative analysis of inflammatory cell infiltration: CD45, CD68 (Mph: macrophages), CD8 and CD3 (T cells), CD20 and CD138 (B cells and plasma cells), counting of positive cells was performed in 10 high power fields (≙ 0.16 mm^2^). c-e: Gene transcript analyses for eosinophilic marker *CCL5* (c), as well as macrophage markers *CD68* (d) and *SIGLEC1/CD169* (e). Expression levels showed significant overexpression of *CCL5* in comparison with NDC and IMNM patients, furthermore, *CD68* and *CD169* are upregulated compared to NDC.


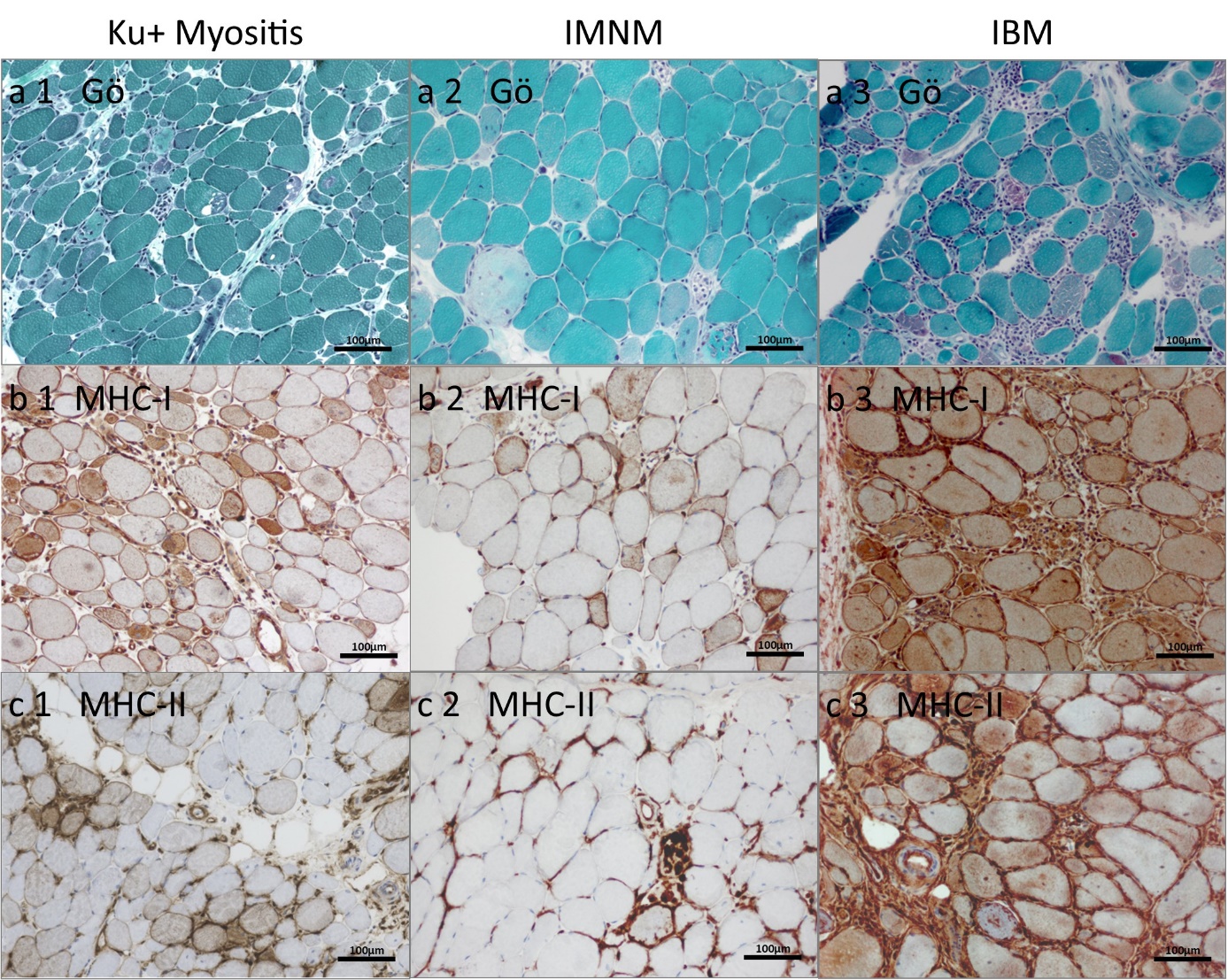


**Supplementary Fig. 2** **Comparison of histopathological and immunohistochemical patterns of Ku+ myositis with immune-mediated necrotizing myopathy (IMNM) and inclusion body myositis (IBM)**

In the comparison of Ku+ myositis with IMNM and IBM the extent of necrotic fibers was more pronounced in Ku+ myositis and the overall numbers of inflammatory infiltrates was also more prominent in Ku+ patients compared to IMNM but less compared with IBM (a). Furthermore, MHC-class I staining is seen in all three entities with IMNM < Ku+ Myositis < IBM (b), while MHC-class II does not stain on myofibers in IMNM but is positive in most cases of Ku+ patients, and constantly positive in IBM (c).


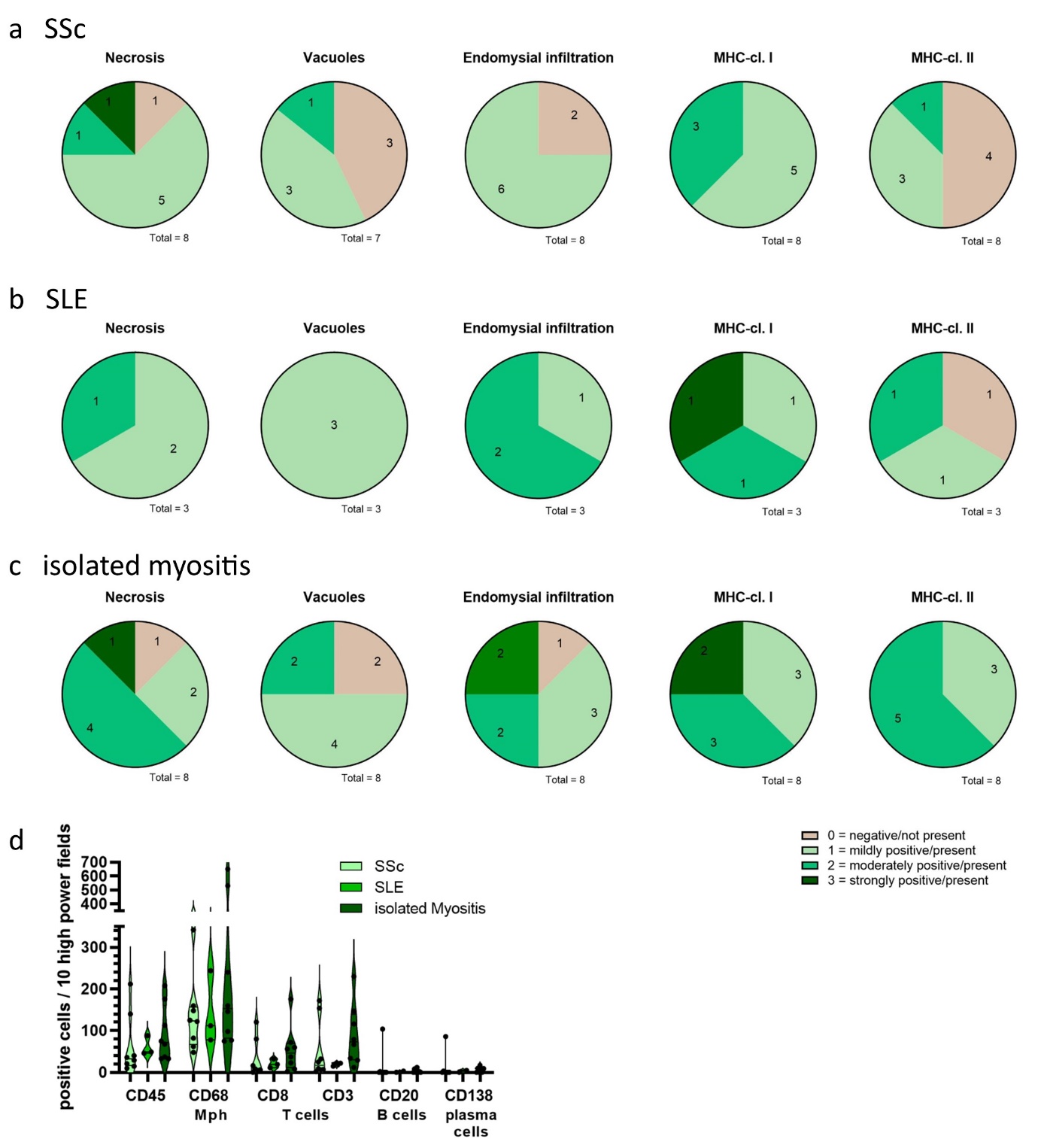


**Supplementary Fig. 3 Comparison of histomorphological scoring aspects of Ku+ biopsies according to clinical phenotype**

Semiquantitative scoring of the histomorphological aspects revealed more pronounced endomysial infiltration and more frequent MHC-class II expression in isolated myositis compared to systemic sclerosis (SSc)- and systemic lupus erythematosus (SLE)-overlap biopsies (a-c). Quantitative analysis of infiltrating cells showed also higher number of macrophages and T cells in isolated myositis (d).


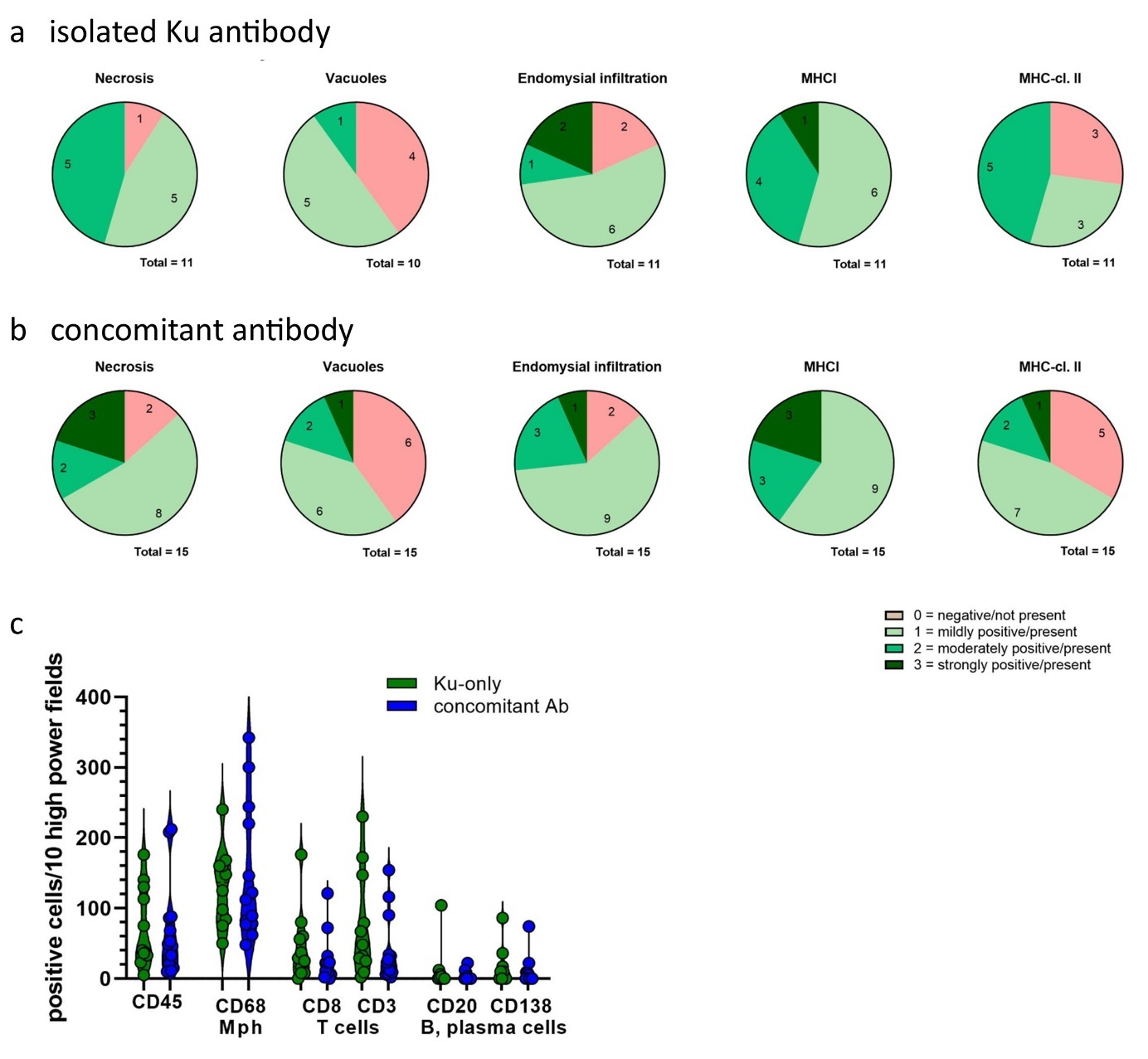


**Supplementary Fig. 4 Comparison of histomorphological scoring aspects of biopsies from isolated Ku+ patients and patients with concomitant antibodies**

Semiquantitative scoring of the histomorphological aspects did not reveal significant differences between the subgroups.

**
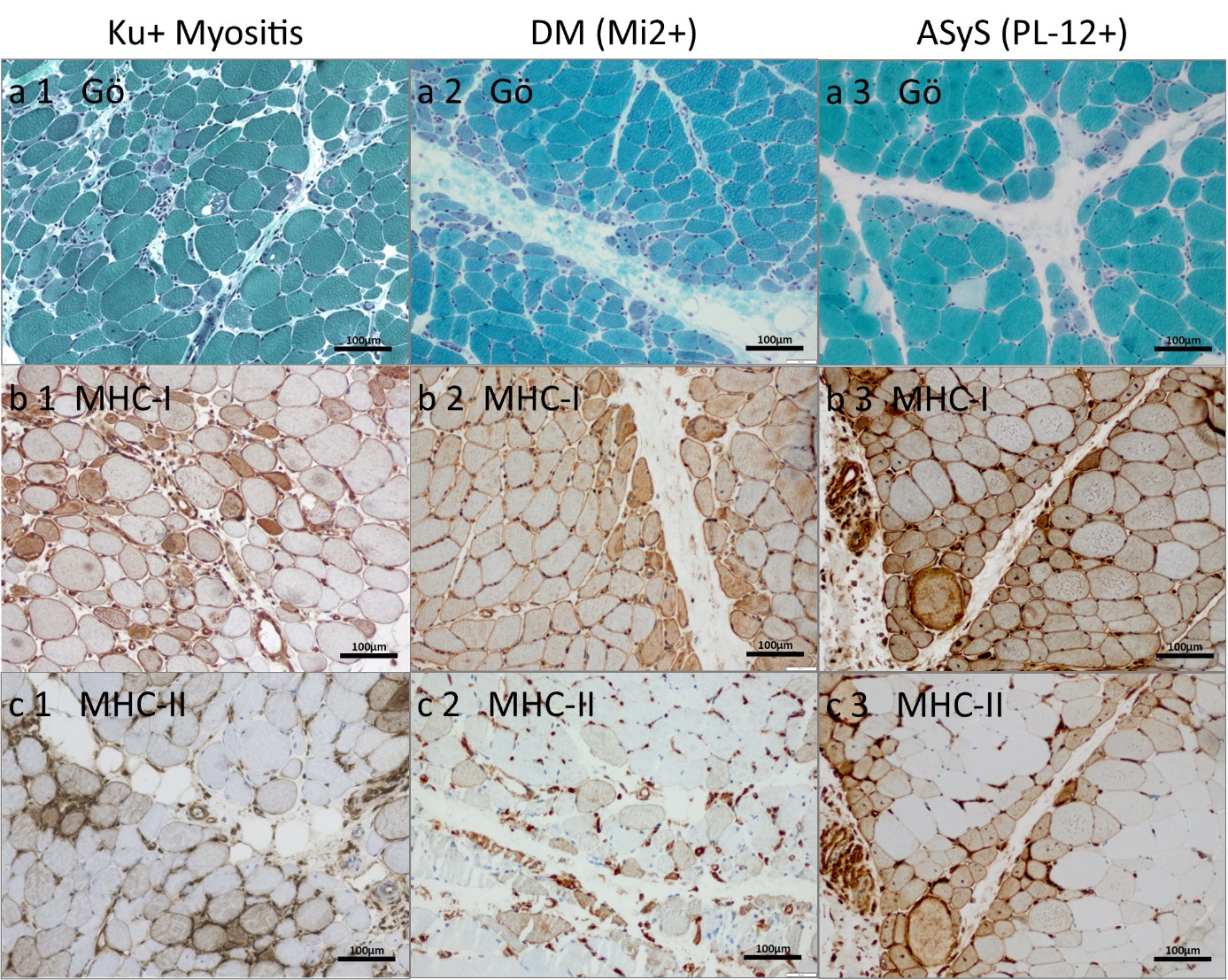
**

**Supplementary Fig. 5** **Comparison of histopathological and immunohistochemical patterns of Ku+ myositis with dermatomyositis (DM) and antisynthetase syndrome (ASyS)**

In the comparison of Ku+ myositis with DM and ASyS we did see more myofiber necrosis and endomysial lymphocyte infiltration as well as sarcolemmal (non-rimmed) vacuoles in the Ku biopsies (a). Furthermore, the MHC-cl. I and MHC-cl. II positivity showed a diffuse, focally enhanced pattern (around areas of endomysial lymphomonocytic infiltration), whereas in DM and ASyS there is a clear and prominent perifascicular gradient for MHC-cl. I (b2, b3) which is also visible with perifascicular enhancement for MHC cl. II in ASyS biopsies (c3) and single /occasional positive fibers in perifascicular regions in the case of Mi2+ DM (c2).


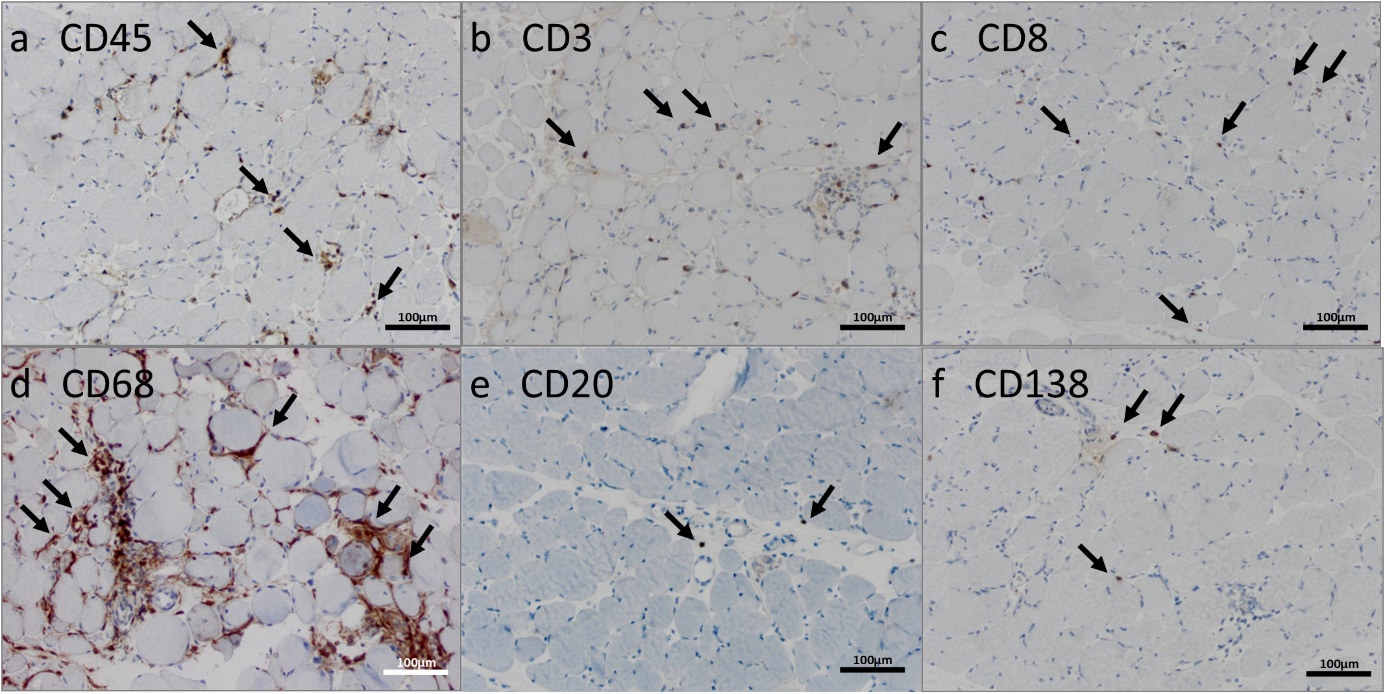


**Supplementary Fig. 6 Inflammatory infiltration in a muscle biopsy sample of a representative Ku+ patient**

Representative figures of inflammatory infiltrates displaying CD45+ leukocytes (a), CD3+ (b), CD8+ (c) T-cells as well as CD68+ macrophages (d), CD20 (e) and CD138 (f) B and plasma cells.

**
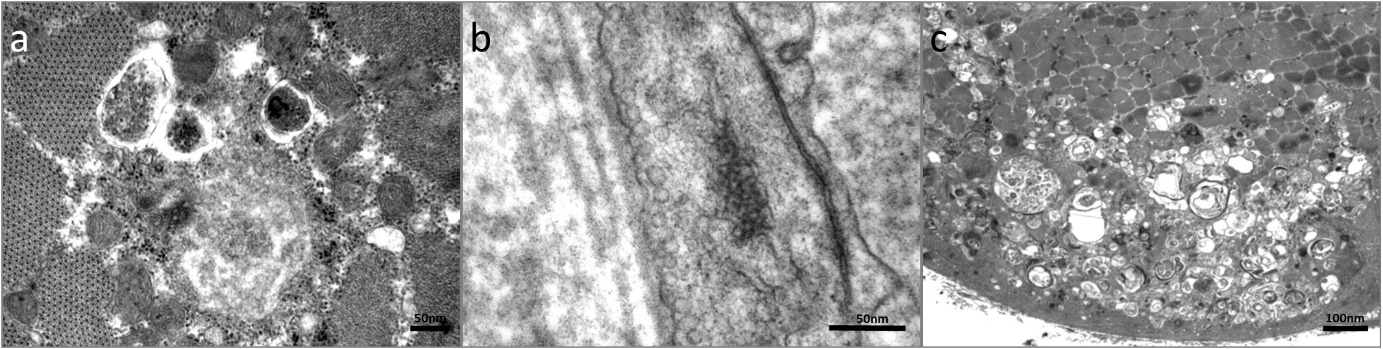
Supplementary Fig. 7 Ultrastructural analysis of vacuoles (a), tubuloreticular inclusions (b) and aggregates (c)**

The ultrastructural analysis revealed that vacuoles of Ku+ myositis did not show rims of deposited debris at the margins, as they do in IBM muscle biopsies (a). Furthermore, occasional tubuloreticular inclusions in endothelial cells of vessel walls could be detected (b). In addition to the filamentous aggregation shown in the main illustration, many of the aggregates show deposition of myelin debris and autophagolysosomal compartments (c). Of note, we never identified tubulofilaments that are characteristic of IBM ultrastructural pathology in myonuclei or in the cytoplasm.

**Supplementary Table 1 Proteomic data filtered for ‘autophagy and cell stress’ -associated proteins (n=48) in mild Ku+ patients, severe Ku+ patients and NDC**

Comparison is performed for severe vs. mild Ku+ patients, severe Ku+ patients vs. NDC and mild Ku+ patients vs. NDC. Some proteins were not detected in NDC (marked in red), and only some changes reach significance (significant *p*-values marked in green).

|  |  |  | **Unique peptides** | | | **Mild/NDC** | | **Severe/NDC** | | **Severe/Mild** | |
| --- | --- | --- | --- | --- | --- | --- | --- | --- | --- | --- | --- |
| **PG.Protein**  **Accessions** | **PG.Genes** | **PG.ProteinDescriptions** | **Severe** | **mild** | **NDC** | **fold-change** | ***p*-value** | **fold-change** | ***p*-value** | **fold-change** | ***p*-value** |
| A4UGR9 | XIRP2 | Xin actin-binding repeat-containing protein 2 | 81 | 20 | 16 | 1,35 | 0,468 | 9,82 | 0,187 | 9,82 | 0,020 |
| Q9BUJ2 | HNRNPUL1 | Heterogeneous nuclear ribonucleoprotein U-like protein 1 | 5 | 2 | 0 |  |  |  |  | 7,14 | 0,024 |
| P13473 | LAMP2 | Lysosome-associated membrane glycoprotein 2 | 2 | 2 | 3 | 1,08 | 0,839 | 2,97 | 0,056 | 4,32 | 0,001 |
| P55795 | HNRNPH2 | Heterogeneous nuclear ribonucleoprotein H2 | 3 | 3 | 2 | 1,16 | 0,451 | 3,9 | 0,171 | 4,15 | 0,022 |
| P07910 | HNRNPC | Heterogeneous nuclear ribonucleoproteins C1/C2 | 7 | 6 | 5 | 1,35 | 0,244 | 5,42 | 0,186 | 4,14 | 0,020 |
| Q9UL46 | PSME2 | Proteasome activator complex subunit 2 | 16 | 13 | 10 | 2,87 | 0,069 | 9,64 | 0,105 | 3,98 | 0,023 |
| P09936 | UCHL1 | Ubiquitin carboxyl-terminal hydrolase isozyme L1 | 11 | 8 | 3 | 3,45 | 0,223 | 9,36 | 0,042 | 3,57 | 0,032 |
| P11279 | LAMP1 | Lysosome-associated membrane glycoprotein 1 | 4 | 2 | 2 | 1,51 | 0,224 | 4,34 | 0,134 | 3,42 | 0,028 |
| Q9UJY1 | HSPB8 | Heat shock protein beta-8 | 7 | 6 | 5 | 1,39 | 0,069 | 3,59 | 0,108 | 3,42 | 0,014 |
| P61289 | PSME3 | Proteasome activator complex subunit 3 | 2 | 1 | 1 | 1,34 | 0,458 | 2,81 | 0,150 | 3,35 | 0,016 |
| Q99729 | HNRNPAB | Heterogeneous nuclear ribonucleoprotein A/B | 4 | 3 | 3 | 1,98 | 0,003 | 5,64 | 0,120 | 3,25 | 0,016 |
| P51148 | RAB5C | Ras-related protein Rab-5C | 4 | 3 | 1 |  |  |  |  | 3,17 | 0,031 |
| Q06323 | PSME1 | Proteasome activator complex subunit 1 | 14 | 10 | 7 | 2,32 | 0,107 | 6,15 | 0,074 | 2,95 | 0,020 |
| Q13501 | SQSTM1 | Sequestosome-1 | 6 | 5 | 4 | 1,25 | 0,329 | 2,82 | 0,161 | 2,78 | 0,020 |
| Q14103 | HNRNPD | Heterogeneous nuclear ribonucleoprotein D0 | 9 | 6 | 6 | 1,09 | 0,439 | 2,43 | 0,125 | 2,77 | 0,011 |
| O95757 | HSPA4L | Heat shock 70 kDa protein 4L | 2 | 1 | 0 |  |  |  |  | 2,74 | 0,044 |
| P52272 | HNRNPM | Heterogeneous nuclear ribonucleoprotein M | 15 | 8 | 8 | 1,35 | 0,006 | 3,43 | 0,166 | 2,66 | 0,019 |
| P09651 | HNRNPA1 | Heterogeneous nuclear ribonucleoprotein A1 | 15 | 13 | 13 | 1,3 | 0,008 | 3,23 | 0,166 | 2,65 | 0,017 |
| Q00839 | HNRNPU | Heterogeneous nuclear ribonucleoprotein U | 20 | 15 | 10 | 1,1 | 0,329 | 2,92 | 0,194 | 2,62 | 0,025 |
| P20340 | RAB6A | Ras-related protein Rab-6A | 5 | 3 | 5 | 1,08 | 0,687 | 2,06 | 0,279 | 2,56 | 0,030 |
| P31943 | HNRNPH1 | Heterogeneous nuclear ribonucleoprotein H | 9 | 7 | 5 | 1,33 | 0,017 | 3,00 | 0,132 | 2,52 | 0,016 |
| P14866 | HNRNPL | Heterogeneous nuclear ribonucleoprotein L | 12 | 8 | 4 | 0,91 | 0,194 | 1,52 | 0,383 | 2,5 | 0,015 |
| Q7Z6Z7 | HUWE1 | E3 ubiquitin-protein ligase HUWE1 | 19 | 9 | 9 | 1,2 | 0,073 | 2,62 | 0,15 | 2,43 | 0,022 |
| P51991 | HNRNPA3 | Heterogeneous nuclear ribonucleoprotein A3 | 11 | 9 | 9 | 1,37 | 0,005 | 2,80 | 0,119 | 2,4 | 0,013 |
| P07339 | CTSD | Cathepsin D | 15 | 13 | 12 | 1,23 | 0,363 | 3,29 | 0,114 | 2,4 | 0,011 |
| P61106 | RAB14 | Ras-related protein Rab-14 | 9 | 5 | 3 | 1,22 | 0,213 | 2,80 | 0,192 | 2,39 | 0,032 |
| Q702N8 | XIRP1 | Xin actin-binding repeat-containing protein 1 | 68 | 52 | 46 | 1,4 | 0,330 | 3,37 | 0,113 | 2,35 | 0,021 |
| P31942 | HNRNPH3 | Heterogeneous nuclear ribonucleoprotein H3 | 4 | 4 | 3 | 1,28 | 0,054 | 2,81 | 0,133 | 2,34 | 0,013 |
| Q1KMD3 | HNRNPUL2 | Heterogeneous nuclear ribonucleoprotein U-like protein 2 | 7 | 3 | 4 | 1,16 | 0,213 | 3,61 | 0,107 | 2,33 | 0,011 |
| O14979 | HNRNPDL | Heterogeneous nuclear ribonucleoprotein D-like | 4 | 3 | 2 | 0,83 | 0,178 | 2,11 | 0,126 | 2,32 | 0,014 |
| P52597 | HNRNPF | Heterogeneous nuclear ribonucleoprotein F | 5 | 2 | 2 | 1,29 | 0,344 | 3,59 | 0,201 | 2,28 | 0,037 |
| Q14157 | UBAP2L | Ubiquitin-associated protein 2-like | 4 | 2 | 2 | 1,04 | 0,882 | 2,45 | 0,062 | 2,25 | 0,006 |
| P22626 | HNRNPA2B1 | Heterogeneous nuclear ribonucleoproteins A2/B1 | 15 | 12 | 13 | 1,36 | 0,007 | 2,83 | 0,167 | 2,25 | 0,023 |
| P08238 | HSP90AB1 | Heat shock protein HSP 90-beta | 44 | 40 | 37 | 1,11 | 0,385 | 2,53 | 0,171 | 2,24 | 0,018 |
| Q96QK1 | VPS35 | Vacuolar protein sorting-associated protein 35 | 10 | 8 | 8 | 1,08 | 0,694 | 2,2 | 0,167 | 2,23 | 0,012 |
| Q9UBQ0 | VPS29 | Vacuolar protein sorting-associated protein 29 | 3 | 3 | 2 | 1,6 | 0,029 | 4,09 | 0,04 | 2,22 | 0,023 |
| Q9BRG1 | VPS25 | Vacuolar protein-sorting-associated protein 25 | 3 | 3 | 2 | 1,45 | 0,070 | 2,83 | 0,012 | 2,17 | 0,001 |
| P43243 | MATR3 | Matrin-3 | 10 | 8 | 9 | 1,11 | 0,236 | 2,36 | 0,163 | 2,13 | 0,018 |
| P55072 | VCP | Transitional endoplasmic reticulum ATPase | 47 | 40 | 34 | 1,06 | 0,606 | 2,09 | 0,108 | 2,12 | 0,01 |
| P61086 | UBE2K | Ubiquitin-conjugating enzyme E2 K | 3 | 2 | 4 | 0,92 | 0,636 | 1,98 | 0,357 | 2,1 | 0,021 |
| Q5T4S7 | UBR4 | E3 ubiquitin-protein ligase UBR4 | 20 | 9 | 9 | 1,31 | 0,164 | 2,52 | 0,09 | 2,09 | 0,014 |
| Q9H0U4 | RAB1B | Ras-related protein Rab-1B | 9 | 8 | 7 | 1,08 | 0,438 | 2,22 | 0,208 | 2,07 | 0,04 |
| O43390 | HNRNPR | Heterogeneous nuclear ribonucleoprotein R | 5 | 3 | 1 | 2,09 | 0,192 | 2,99 | 0,031 | 2,05 | 0,025 |
| Q14108 | SCARB2 | Lysosome membrane protein 2 | 4 | 3 | 2 | 1,4 | 0,045 | 3,56 | 0,011 | 2,02 | 0,004 |
| Q6ZT12 | UBR3 | E3 ubiquitin-protein ligase UBR3 | 5 | 2 | 5 | 0,8 | 0,286 | 1,94 | 0,085 | 2,01 | 0,008 |
| P20339 | RAB5A | Ras-related protein Rab-5A | 5 | 3 | 4 | 1,22 | 0,066 | 2,01 | 0,050 | 2,01 | 0,01 |
| Q13148 | TARDBP | TAR DNA-binding protein 43 | 5 | 5 | 3 | 1,23 | 0,237 | 2,14 | 0,148 | 2,01 | 0,032 |
| 07900 | HSP90AA1 | Heat shock protein HSP 90-alpha | 22 | 21 | 16 | 0,85 | 0,060 | 1,66 | 0,405 | 2,00 | 0,050 |

**Supplementary Table 2 Antibodies used for morphological analysis**

| **Antibody** | **Company** | **dilution** | **species** |
| --- | --- | --- | --- |
| aB-Crystallin | Abcam, ab13496 | 1:2500 | mouse |
| BAG3 | Abcam, ab47124 | 1:100 | rabbit |
| beta (ß)-5i | Enzo, LMP7-1 | 1:100 | mouse |
| CD3 | DAKO, A0452 | 1:100 | rabbit |
| CD8 | DAKO, M7050 | 1:100 | mouse |
| CD20 | DAKO, M0755 | 1:200 | mouse |
| CD31 | DAKO, M0823 | 1:100 | mouse |
| CD45 | DAKO, M0701 | 1:400 | mouse |
| CD68 | DAKO, M0718 | 1:100 | mouse |
| CD138 | DAKO, M7228 | 1:30 | mouse |
| CD169/Siglec1 | Novus Biologicals, NB600-534 | 1:200 | mouse |
| C5b-9 | DAKO, M0777 | 1:200 | mouse |
| Desmin | DAKO, M0760 | 1:100 | mouse |
| HSP70 | Abcam, ab6535 | 1:100 | mouse |
| ISG15 | Abcam, ab14374 | 1:100 | rabbit |
| LC3 | Nanotools Art, 0260-100 | 1:50 | mouse |
| MHC-class I | DAKO, M0736 | 1:1.000 | mouse |
| MHC-class II | DAKO, M0775 | 1:100 | mouse |
| MHC-neonatal | Novacastra, NCL-MHCn | 1:20 | mouse |
| MHC-developmental | Novocastra, NCL-MHCd | 1:5 | mouse |
| Myotilin | Novocastra, NCL-MYOTILIN | 1:20 | mouse |
| MxA | Millipore, MABF938 | 1:100 | mouse |
| p62 / SQSTM1 | BD, 610832 | 1:100 | mouse |

**Supplementary Table 3: Subgroup analysis of clinical data comparing Ku+ myositis patients with isolated Ku antibodies and concomitant antibodies**

In the group of isolated Ku antibodies, there were 3 SSc patients, one SjS patient, 5 patients diagnosed as myositis and 2 patients diagnosed with RA. In the cohort of concomitant antibodies, 5 patients were diagnosed with SSc, 2 with SjS, 3 with SLE, 3 with isolated myositis and 3 with RA. Patients in the isolated Ku-antibody group tend to have more sclerodactyly but less dysphagia, whereas cardiac involvement was similar in both groups.

| % (n/reported) | All  (n=26) | Isolated Ku-antibody (n=11) | Ku and concomitant antibody (n=15) |
| --- | --- | --- | --- |
| Muscle Weakness | 54.5% (12/22) | 40.0% (4/10) | 66.7% (8/12) |
| Arthralgia | 80.0% (16/20) | 75.0% (6/8) | 83.3% (10/12) |
| Raynaud‘s phenomenon | 63.6% (14/22) | 55.6% (5/9) | 69.2% (9/13) |
| Sclerodactyly | 38.1% (8/21) | 57.1% (4/7) | 28.6% (4/14) |
| Dermatomyositis-like skin findings | 33.3% (8/24) | 44.4% (4/9) | 33.3% (5/15) |
| Dysphagia | 58.8% (10/17) | 42.9% (3/7) | 70% (7/10) |
| ILD | 38.1% (8/21) | 44.4% (4/9) | 33.3% (4/12) |
| PAH | 33.3% (6/18) | 42.9% (3/7) | 27.3% (3/11) |
| Renal Involvement | 23.1% (3/13) | 25% (1/4) | 22.2% (2/9) |
| Cardiac Involvement | 61.9% (14/21) | 66.7% (6/9) | 66.7% (8/12) |
| Neuropathy | 52.6% (10/19) | 57.1% (4/7) | 50% (6/12) |
| Creatine kinase mean  (<145U/l standard range) | 1772 U/l | 1340 U/l | 2105 U/l |

**Supplementary Table 4: Comparison of proteomic analysis in Ku+ myositis, immune mediated necrotizing myopathy (IMNM) and inclusion body myositis (IBM) compared to non-disease controls (NDC)**

The top 10 upregulated autophagy and cell stress proteins in Ku+ myositis compared to NDC are also displayed in figure 2g. Here we additionally present the regulation of these proteins in IMNM and IBM compared to NDC (HNRNPUL1 showed no expression in NDC and therefore no fold-change ratio could be calculated), showing partly upregulation of XIRP2 and PSME2 in IMNM, but the other proteins were not upregulated and often not expressed. Furthermore, there is no up-regulation of these proteins in IBM. N.d. = no detected expression of this protein.

|  |  | **Mild/NDC** | **Severe/NDC** | **IMNM/NDC** | **IBM/NDC** |
| --- | --- | --- | --- | --- | --- |
| **PG.Gene** |  | **fold-change** | **fold-change** | **fold-change** | **fold-change** |
| XIRP2 | Xin actin-binding repeat-containing protein 2 | 1.35 | 9.82 | 10.08 | 0.95 |
| HNRNPUL1 | Heterogeneous nuclear ribonucleoprotein U |  |  |  |  |
| LAMP2 | Lysosome-associated membrane glycoprotein 2 | 1.08 | 2.97 | n.d. | n.d. |
| HNRNPH2 | Heterogeneous nuclear ribonucleoprotein H2 | 1.16 | 3.90 | 1.73 | n.d. |
| HNRNPC | Heterogeneous nuclear ribonucleoproteins C1/C2 | 1.35 | 5.42 | n.d. | 1.03 |
| PSME2 | Proteasome activator complex subunit 2 | 2.87 | 9.64 | 8.30 | 1.23 |
| UCHL1 | Ubiquitin carboxyl-terminal hydrolase isozyme L1 | 3.45 | 9.36 | n.d. | 1.55 |
| LAMP1 | Lysosome-associated membrane glycoprotein 1 | 1.51 | 4.34 | n.d. | n.d. |
| HSPB8 | Heat shock protein beta-8 | 1.39 | 3.59 | n.d. | 2.00 |
| PSME3 | Proteasome activator complex subunit 3 | 1.34 | 2.81 | n.d. | 1.91 |
